# Is there enough evidence supporting the clinical adoption of clear cell likelihood score (ccLS)? An updated systematic review and meta-analysis

**DOI:** 10.1186/s13244-024-01829-y

**Published:** 2024-10-09

**Authors:** Jingyu Zhong, Yangfan Hu, Yue Xing, Xianwei Liu, Xiang Ge, Yibin Wang, Yuping Shi, Junjie Lu, Jiarui Yang, Yang Song, Minda Lu, Jingshen Chu, Huan Zhang, Defang Ding, Weiwu Yao

**Affiliations:** 1 Department of Imaging, Tongren Hospital, Shanghai Jiao Tong University School of Medicine, Shanghai, 200336 China; 2 Department of Urology, Tongren Hospital, Shanghai Jiao Tong University School of Medicine, Shanghai, 200336 China; 3 Department of Nephrology, Tongren Hospital, Shanghai Jiao Tong University School of Medicine, Shanghai, 200336 China; 4 Department of Epidemiology and Population Health, Stanford University School of Medicine, Stanford, CA 94305 USA; 5 Department of Biomedical Engineering, Boston University, Boston, MA 02215 USA; 6 MR Research Collaboration Team, Siemens Healthineers Ltd., Shanghai, 200126 China; 7 MR Application, Siemens Healthineers Ltd., Shanghai, 200126 China; 8 Department of Science and Technology Development, Ruijin Hospital, Shanghai Jiao Tong University School of Medicine, Shanghai, 200025 China; 9 Department of Radiology, Ruijin Hospital, Shanghai Jiao Tong University of Medicine, Shanghai, 200025 China

**Keywords:** Carcinoma (renal cell), Magnetic resonance imaging, Tomography (X-ray computed), Diagnosis

## Abstract

**Objective:**

To review the evidence for clinical adoption of clear cell likelihood score (ccLS) for identifying clear cell renal cell carcinoma (ccRCC) from small renal masses (SRMs).

**Methods:**

We distinguished the literature on ccLS for identifying ccRCC via systematic search using PubMed, Embase, Web of Science, China National Knowledge Infrastructure, and Wanfang Data until 31 March, 2024. The risk of bias and concern on application was assessed using the modified quality assessment of diagnostic accuracy studies (QUADAS-2) tool. The level of evidence supporting the clinical adoption of ccLS for identifying ccRCC was determined based on meta-analyses.

**Results:**

Eight MRI studies and three CT studies were included. The risk of bias and application were mainly related to the index test and flow and timing, due to incomplete imaging protocol, unclear rating process, and inappropriate interval between imaging and surgery. The diagnostic odds ratios (95% confidence intervals) of MRI and CT ccLS were 14.69 (9.71–22.22; 6 studies, 1429 SRM, 869 ccRCC), and 5.64 (3.34–9.54; 3 studies, 296 SRM, 147 ccRCC), respectively, for identifying ccRCC from SRM. The evidence level for clinical adoption of MRI and CT ccLS were both rated as weak. MRI ccLS version 2.0 potentially has better diagnostic performance than version 1.0 (1 study, 700 SRM, 509 ccRCC). Both T2-weighted-imaging with or without fat suppression might be suitable for MRI ccLS version 2.0 (1 study, 111 SRM, 82 ccRCC).

**Conclusion:**

ccLS shows promising diagnostic performance for identifying ccRCC from SRM, but the evidence for its adoption in clinical routine remains weak.

**Critical relevance statement:**

Although clear cell likelihood score (ccLS) demonstrates promising performance for detecting clear cell renal cell carcinoma, additional evidence is crucial to support its routine use as a tool for both initial diagnosis and active surveillance of small renal masses.

**Key Points:**

- Clear cell likelihood score is designed for the evaluation of small renal masses.
- Both CT and MRI clear cell likelihood scores are accurate and efficient.
- More evidence is necessary for the clinical adoption of a clear cell likelihood score.

**Graphical Abstract:**

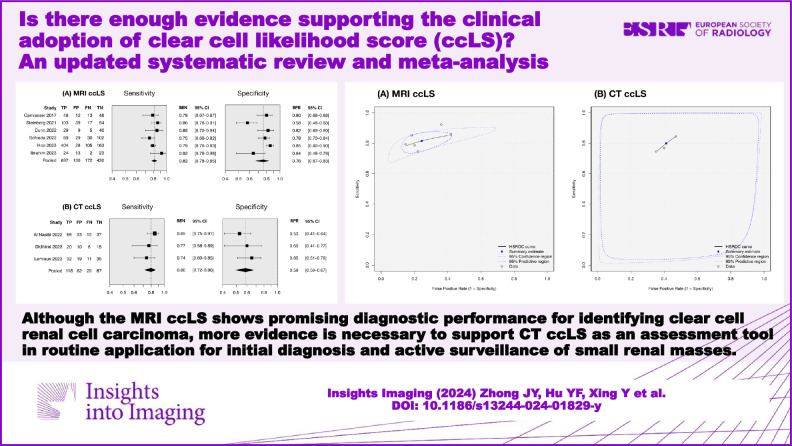

## Introduction

Small renal masses (SRM) are defined as renal masses with ≥ 25% of the lesion exhibiting enhancement and ≤ 4 cm in diameter [1–5]. Over 70% of SRM are malignant, with the most common subtype being clear cell renal cell carcinoma (ccRCC) that needs further management [6, 7]. The detection of SRM is becoming more common with the increasing use of cross-sectional imaging. Up to 70% of SRM are detected incidentally for an unrelated medical condition [8]. Early detection of SRM allows early intervention, but it does not improve the cancer-specific mortality in this situation [9, 10]. This indicated that the diagnosis of SRM does not always translate to a true survival benefit. The management of the SRM should balance the possibility of malignancy and nephron loss due to therapeutic interventions. A careful selection of cases using cross-sectional imaging modalities is necessary before clinical decision-making. The clear cell renal carcinoma likelihood score (ccLS) algorithm is designed to better identify ccRCC from other subtypes of renal carcinoma with less invasive behavior and benign lesions [11–13]. This tool aims to better identify cases that can be safely surveilled rather than immediate resection or ablation, hereby minimizing unnecessary interventions and preserving renal function.

The MRI ccLS algorithm is a standardized five-tiered Likert score algorithm offering a likelihood of SRM to be a ccRCC from “very unlikely” to “very likely” categories and is useful in non-invasively identifying the clear cell subtype [14–17]. The MRI ccLS is derived from multiparametric MRI, incorporating a series of imaging features, including T2 signal intensity, microscopic fat, corticomedullary phase enhancement, segmental enhancement inversion, arterial-to-delayed enhancement ratio, and DWI signal intensity (Supplementary Fig. S1). By assigning an MRI ccLS, patients can be stratified into those who may or may not benefit from further treatment. A systematic review and meta-analysis declared that the MRI ccLS algorithm has moderate to high accuracy for identifying ccRCC from SRM with a moderate inter-reader agreement [18]. Unfortunately, the scientific robustness of this review is limited due to the inclusion of overlapping cohorts in the meta-analysis [19–21]. Consequently, a re-evaluation of evidence should be conducted on the MRI ccLS algorithm before the clinical adoption. Furthermore, since its initial publication, the MRI ccLS algorithm has undergone several modifications [19]. It remains unclear whether the different versions of ccLS impact their ability to accurately identify ccRCC [20–26].

Recently, a new CT ccLS algorithm has been developed to allow a more frequent and convenient evaluation of SRM, specifically targeting the prediction of ccRCC likelihood [27]. This CT ccLS algorithm follows the same five-tiered Likert score format as the MRI ccLS and is derived from contrast-enhanced CT scans, utilizing the mass-to-cortex corticomedullary attenuation ratio and heterogeneity score (Supplementary Fig. S2). Although the CT ccLS algorithm has been validated in two additional centers [28, 29], a comprehensive assessment of the available evidence has yet to be conducted. It is essential to evaluate and compare the diagnostic performance and inter-reader agreement of both the CT and MRI ccLS algorithms to determine their respective strengths and limitations in clinical practice.

Therefore, we decided to perform an update on this systematic review and meta-analysis [30]. This study aimed to review the evidence for the clinical adoption of MRI and CT ccLS algorithms for identifying ccRCC from SRM.

## Methods

### Study protocol and workflow

We conducted and reported the systematic review according to the Preferred Reporting Items for Systematic Reviews and Meta-Analyses (PRISMA) statements [31–34]. We have drafted a protocol before the systematic review and prospectively registered it via PROSPERO, the International Prospective Register of Systematic Reviews (https://www.crd.york.ac.uk), with an identifier of CRD42024527042 (Supplementary Note S1). The literature search, study selection, data extraction, and quality assessment were performed and validated by two of five radiologists who have 6 to 10 years of experience, respectively. Any disagreements during these stages were solved with discussions or consultation with the review group. Our review group comprises health professionals with diverse backgrounds and experiences.

### Literature search and study selection

We conducted a search of PubMed, Embase, Web of Science, China National Knowledge Infrastructure, and Wanfang Data to distinguish the literature on the use of ccLS algorithms for identifying ccRCC from SRM until 31 March, 2024. The publication year of the literature should be after 2017, when the ccLS had been proposed [19]. We restricted the search to publications in English, Chinese, Japanese, German, and French. After the removal of duplications, the titles and abstracts of the literature were screened. The full-texts and supplementary materials of the literature were obtained to decide whether they were eligible. The reference list of the included literature and relevant reviews were browsed to identify extra potentially eligible literature. The details of the search strategy and study selection process are outlined (Supplementary Note S2).

### Data extraction and quality assessment

We used a data collection sheet to standardize the detection and record of bibliographical information, study characteristics, methodological details, and diagnostic performance metrics of included studies (Supplementary Table S1). The included studies were assessed using the modified quality assessment of diagnostic accuracy studies (QUADAS-2) tool [35] with specified signal questions for the current systematic review (Supplementary Table S2). The items discussed during data extraction and quality assessment are recorded (Supplementary Note S3).

### Data synthesis and analysis

We conducted meta-analysis using R language version 4.2.1 (https://www.r-project.org/) within RStudio version 1.3.1093 (https://posit.co/) with relevant packages and their corresponding website applications, namely MetaDTA (https://crsu.shinyapps.io/MetaDTA/) [36, 37], and metaumbrella (https://www.metaumbrella.org) [38, 39]. We meta-analyzed the diagnostic performance of MRI and CT ccLS algorithms for identifying ccRCC from SRM, respectively. The two-by-two tables for the diagnostic performance of ccLS algorithms were directly extracted from the literature or reconstructed using available data. The cutoff for the ccRCC was set at 4, i.e., a SRM with a ccLS rating ≥ 4 was considered as ccRCC. The data of overall diagnostic performance were chosen if reported. Otherwise, the data of the rater with worst diagnostic performance were chosen, to provide a conservative estimate for ccLS algorithms. In the case of potentially overlapping studies, we only included the studies with the largest sample size. The bivariate random-effect model was selected to calculate the sensitivity, specificity, and 95% confidence interval (CI) due to the varying characteristics of the included studies. A hierarchical summary receiver operating characteristic (HSROC) curve was plotted to show the diagnostic performance. The following analysis was performed for the evidence-level rating [38]: diagnostic odds ratio (DOR) with 95% CI and corresponding *p-*value using the random-effect model, the heterogeneity estimated by the Higgins *I*^*2*^ statistic, the small study effects, the publication bias assessed by Egger’s and Begg’s test, the 95% prediction interval, and the excess significance bias. The robustness of the meta-analyses was assessed using leave-one-out sensitivity analysis. A two-tailed *p*-value < 0.05 was considered statistically significant, while a two-tailed *p*-value > 0.10 indicated a low publication bias. The level of evidence supporting the clinical adoption of ccLS was determined according to the results of meta-analyses (Supplementary Table S3). The handling of overlapping studies and data analysis details are described (Supplementary Note S4).

## Results

### Study inclusion

**Fig. 1:**
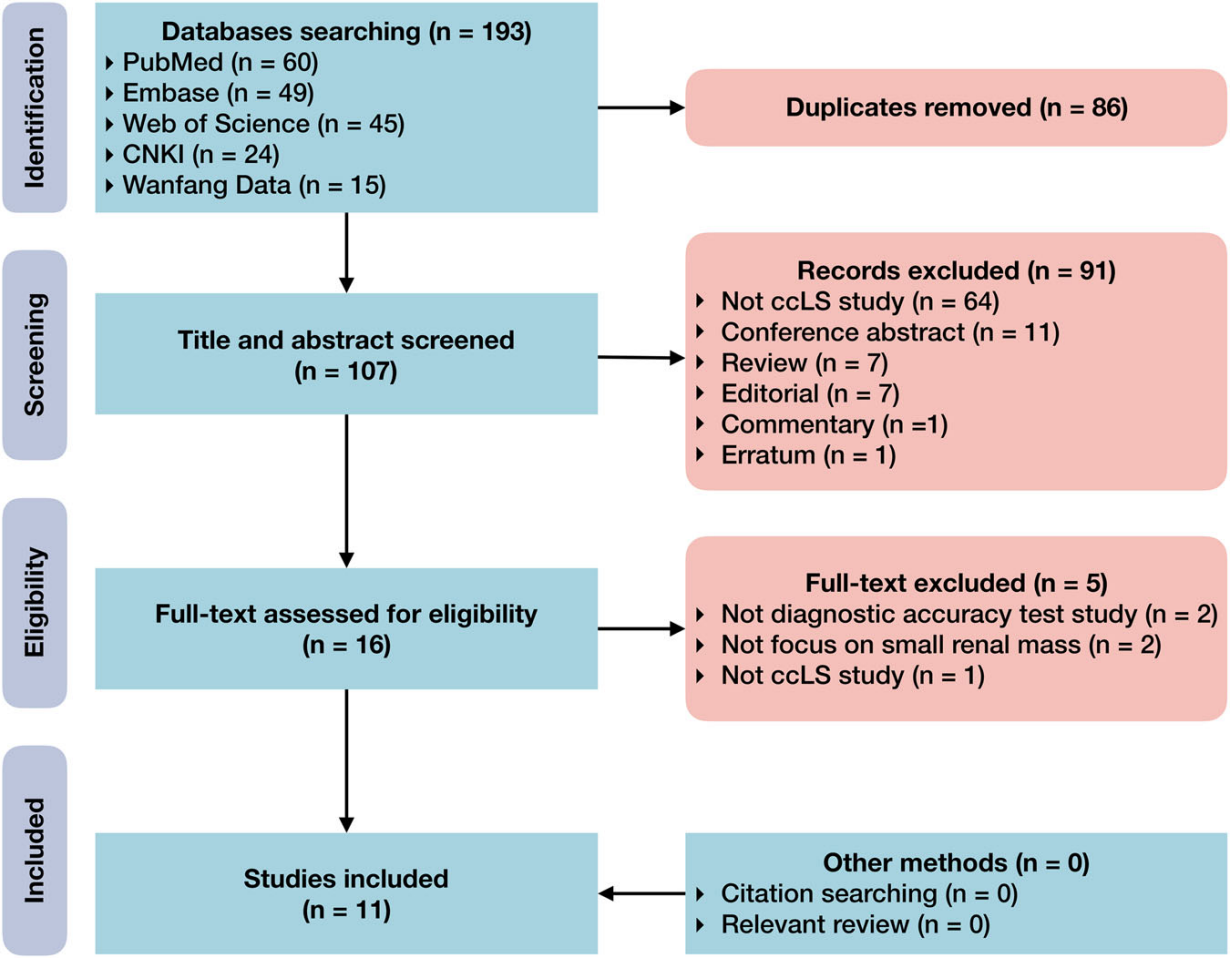
Flowchart of study search and selection. ccLS, clear cell likelihood score; CNKI, China National Knowledge Infrastructure

The systematic search resulted in 193 records. We screened 107 unique records of titles and abstracts after the removal of 86 duplications. The full-texts and supplementary materials of 16 potentially eligible literature were assessed. Finally, 11 studies were included for systematic review (Fig. 1) [19–29]. No extra potentially eligible literature was identified by browsing the reference lists of included studies or relevant reviews. The included studies and excluded full texts with justifications are recorded (Supplementary Note S2).

### Study characteristics

The characteristics of the 11 included studies are summarized (Table 1) [19–29]. The studies were more frequently published in radiological journals (8/11, 73%) [22–24, 26–29, 40]. Most of the studies were conducted retrospectively (9/11, 82%) [19, 22–29] within a single center (8/11, 73%) [19, 20, 22, 24, 26–29] with the modality of MRI (8/11, 73%) [19–26]. The mean ± standard deviation, median (range) of number of patients and percentage of ccRCC were 176.5 ± 191.5, 110.5 (51.0–691.0), and 53.3 ± 11.2, 51.0 (41.0–73.9) %, respectively. The rating was performed by 2.2 ± 1.2, 2 (1.0–5.0) raters per study, applying MRI ccLS algorithm version 2.0 (5/12, 42%) [22–26], MRI ccLS algorithm version 1.0 (4/12, 33%) [19, 20, 25, 40], and CT ccLS algorithm (3/12, 25%) [27–29]. The appropriate blindness method was applied in most of the studies (10/11, 91%), including blindness to histological diagnosis (8/11, 73%) [19, 22–28], or prospectively assigned ccLS rating in a structured radiological report (2/11, 18%) [20, 21]. All the included studies assessed the diagnostic performance of ccLS algorithms. One study compared and suggested that MRI ccLS algorithm version 2.0 potentially had better diagnostic performance than version 1.0 [25]. Another study indicated that both T2-weighted imaging with or without fat suppression were suitable for MRI ccLS algorithm version 2.0 [24]. The details of each included study are summarized (Supplementary Tables S4 to S8).

**Table 1:** Characteristics of included studies

Study characteristics	Data
Journal type (*N* = 11), *n* (%)	
Radiological journal	8 (73)
Clinical journal	3 (27)
Study design (*N* = 11), *n* (%)	
Retrospective	9 (82)
Prospective	2 (18)
Study center (*N* = 11), *n* (%)	
Single center	8 (73)
Multiple centers	3 (27)
Imaging modality (*N* = 11), *n* (%)	
MRI	8 (73)
CT	3 (27)
Number of patients, mean ± standard deviation, median (range)	176.5 ± 191.5, 110.5 (51.0–691.0)
Number of masses, mean ± standard deviation, median (range)	172.5 ± 186.0, 111.0 (51.0–700.0)
Number of ccRCC, mean ± standard deviation, median (range)	102.9 ± 139.0, 61.0 (25.0–509.0)
Percentage of ccRCC, mean ± standard deviation, median (range)	53.3 ± 11.2, 51.0 (41.0–73.9)
Number of raters, mean ± standard deviation, median (range)	2.2 ± 1.2, 2 (1.0–5.0)
ccLS algorithm (*N* = 12), *n* (%)^a^	
MRI ccLS algorithm version 1.0	4 (33)
MRI ccLS algorithm version 2.0	5 (42)
CT ccLS algorithm	3 (25)
Blindness of ccLS rating (*N* = 11), *n* (%)	
Prospectively assigned ccLS rating	2 (18)
Blinded to histological diagnosis	8 (73)
Not reported	1 (9)

*ccLS* clear cell likelihood score

^a^ One study compared MRI ccLS algorithms version 1.0 and version 2.0

### Study quality

**Fig. 2:**
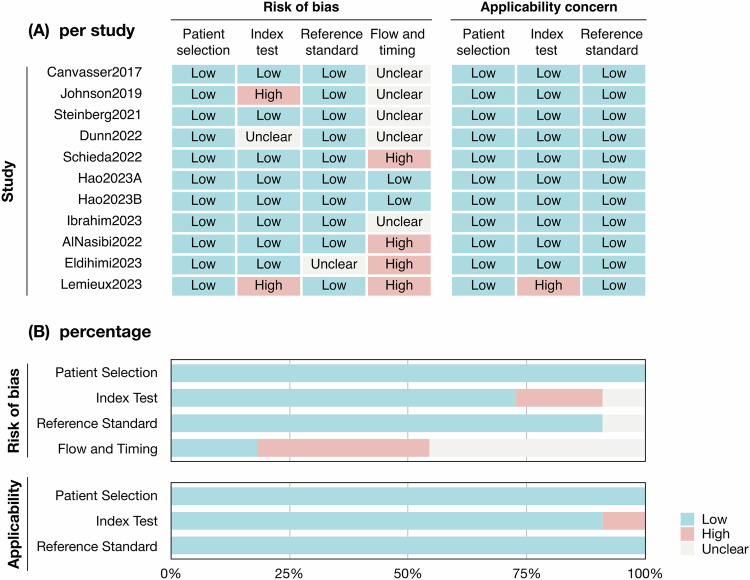
Risk of bias and applicability concerns assessment. **A** The risk of bias and applicability concerns assessment per study; **B** The percentage of the risk of bias and applicability concerns assessment

The results of the risk of bias and applicability concerns assessments are summarized (Fig. 2). The risk of bias and application concerns were mainly related to the index test and flow and timing. For the index test, the MRI protocol has not been fully reported [22], the training process of ccLS was not introduced [20], and the blindness method was not declared [29]. For the flow and timing, the intervals between the imaging and surgery were not reported in five studies [19, 20, 22, 26, 40], while the intervals were considered to be inappropriate in four studies [23, 27–29]. Only one study was rated as unclear risk of bias in reference standard because the histology of biopsy was used as the reference standard [28]. The QUADAS-2 assessment of each study by two reviewers was recorded (Supplementary Table S9).

### Diagnostic performance of MRI and CT ccLS

**Fig. 3:**
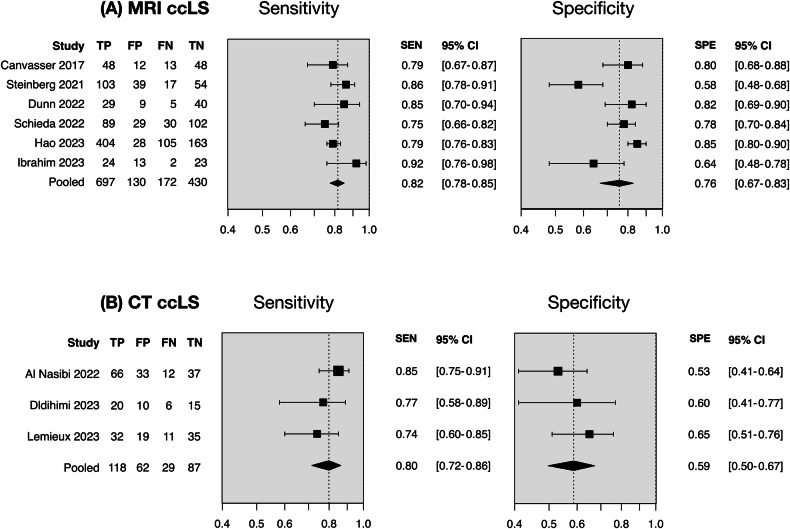
Forest plots of pooled sensitivity and specificity. **A** MRI ccLS algorithm; **B** CT ccLS algorithm. TP = histologically diagnosed clear cell renal carcinoma rated with a ccLS rating ≥ 4, FP = histologically diagnosed non-clear cell renal carcinoma rated with a ccLS rating ≥ 4, FN = histologically diagnosed clear cell renal carcinoma rated with a ccLS rating ≤ 3, TN = histologically diagnosed non-clear cell renal carcinoma rated with a ccLS rating ≤ 3. ccLS, clear cell likelihood score

**Fig. 4:**
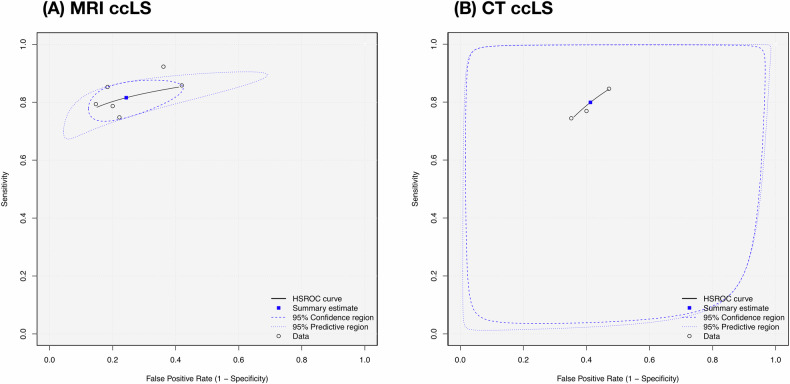
Hierarchical summary receiver operating characteristic curve. **A** MRI ccLS algorithm; **B** CT ccLS algorithm. HSROC, hierarchical summary receiver operating characteristic

The meta-analysis included nine studies [19, 21–24, 26–29] after the exclusion of two overlapping studies [20, 24] (Supplementary Note S4). The two-by-two data were directly extracted or reconstrued for meta-analysis (Supplementary Table S10). The diagnostic odds ratio of six MRI ccLS studies [19, 21–23, 25, 26] and three CT ccLS studies [27–29] were 14.69 (95% CI 9.71–22.22), and 5.64 (95% CI 3.34–9.54), with moderate and low heterogeneity, respectively (Table 2). The high sensitivity and specificity suggested their promising diagnostic performance (Fig. 3). The area under the HSROC curve of MRI and CT ccLS were 0.86 (95% CI 0.82–0.89), and 0.76 (95% CI 0.69–0.82), respectively (Fig. 4). The small study effects, publication, and excess significance were not detected. However, the 95% prediction interval did not exclude the null considering CT ccLS studies. The leave-one-out sensitivity analysis indicated that the results of meta-analyses were robust (Supplementary Table S11). The evidence-level ratings for adopting MRI and CT ccLS algorithms in clinical routine were both weak mainly due to insufficient number of cases.

**Table 2:** Evidence supporting the clinical adoption of ccLS algorithms

Diagnostic performance	MRI ccLS algorithm	CT ccLS algorithm
Sample size		
Number of studies	6	3
Number of tumors	1539	296
Number of ccRCC/non-ccRCC	869/560	147/149
Meta-analysis results		
DOR (95% CI)	14.69 (9.71–22.22)	5.64 (3.34–9.54)
*p*-value for DOR	4.13 × 10^−37^	1.04 × 10^−10^
Sensitivity (95% CI)	0.82 (0.78–0.85)	0.80 (0.72–0.86)
Specificity (95% CI)	0.76 (0.67–0.83)	0.59 (0.50–0.67)
Positive likelihood ratio (95% CI)	3.36 (2.46–4.58)	1.94 (1.56–2.41)
Negative likelihood ratio (95% CI)	0.24 (0.20–0.29)	0.34 (0.24–0.49)
Aera under the curve (95% CI)	0.86 (0.82–0.89)	0.76 (0.69–0.82)
Higgins *I*^2^ test	40.75%	0.00%
Egger’ s test, *p*-value	0.987	0.326
Begg’ s test, *p*-value	0.365	0.305
95% PI excluding the null	Yes	No
DOR of largest study excluding the null	Yes	Yes
Excess significance	No	No
Robustness of results according to leave-one-out analysis	Yes	Yes
Level of evidence according to Ioannidis criteria		
Evidence level	Weak	Weak
Main weakness	Insufficient cases	Insufficient cases

*ccLS* clear cell likelihood score, *ccRCC* clear cell renal cell carcinoma, *CI* confidence interval, *DOR* diagnostic odds ratio

## Discussion

Our study found that both MRI and CT ccLS algorithms have promising diagnostic performance in identifying ccRCC from SRM but were only supported by the weak level of evidence considering their adoption into clinical practice. The incomplete imaging protocol, unclear rating process, and inappropriate interval between imaging and surgery may introduce the risk of bias to the ccLS studies. It is still unclear whether the MRI version of ccLS algorithm and included MRI sequences have a clinically significant impact on the diagnostic performance.

In comparison to a previous systematic review on ccLS algorithms [17], our systematic review included Chinese studies and recently established CT ccLS algorithm studies [24–29]. It is benefited by the inclusion of more studies with samples of different ethnicities and CT modalities, to indicate the eligibility of ccLS algorithms. Meanwhile, we excluded studies with overlapping samples ensure scientific robustness [19–21]; thus, reducing bias and providing results closer to the true diagnostic performance of ccLS algorithms. Further, we confined our meta-analysis to SRM ≤ 4 cm, as per the MRI ccLS algorithm’s proposed use [19]. Unlike a previous meta-analysis [17], we considered that it is not suitable to arbitrarily expand the scope of use without any validation. The size of RCC is an important factor for the selection of surgery procedure [41–43]. The RCC ≤ 4 cm is more frequently treated with partial nephrectomy, while the RCC > 4 cm is preferred to be treated with radical nephrectomy. Nevertheless, it is of interest to investigate whether the ccLS algorithm can identify patients with RCC > 4 cm for whom it is safe to undergo more conservative surgery procedures that can preserve more nephrons.

The results of risk of bias and applicability concerns assessment are quite different between the previous systematic review and ours. Although the specific signal questions of the previous systematic review are not available, we supposed that the difference may be due to the varied questions used for assessment. We believe the index test process should be more clearly declared to evaluate their potential influence on image interpretation [16]. Dunn et al [22] did not provide the standard for MRI protocol; therefore, it is unclear whether there are extra MRI sequences that potentially influence the ccLS evaluation. Johnson et al [20] did not report the training process of ccLS, and Lemieux et al [29] did not declare the blindness of radiologists. Both have unclear impacts on the final rating and are related to the diagnostic performance of ccLS. The flow and timing should also be emphasized since the appearance of the SRM may change if the interval between imaging and surgery is too long [11]. Unfortunately, the intervals between the imaging and surgery were not reported in five studies [19, 20, 22, 26, 40] and were considered to be inappropriate in four studies [23, 27–29]. Only one study used the histology of biopsy [28]. It can be improved if the histology after surgery is available, as in other studies [19–27, 29]. The patient selection was appropriate in all the included ccLS studies. We encourage future studies to validate the ccLS algorithms with an improved design avoiding these disadvantages.

There are several unsolved problems that hinder the clinical adoption of the MRI ccLS algorithm. One major issue is the lack of a standardized MRI protocol, which can significantly impact the algorithm’s interpretation. The different techniques for generating the T1-weighted in-phase and opposed-phase images can influence on the presence of microscopic fat, which has substantial effect on the interpretation of SRM using the MRI ccLS algorithm [44, 45]. Another potential alternative sequence is T2-weighted images with fat saturation. Although the MRI ccLS algorithm recommends a T2-weighted non-fat-saturated images for evaluation, the fat-saturated images may not influence the results [24]. Therefore, T2-weighted images with fat saturation can be accepted as an alternative when non-fat-saturated images are not available. The diffusion-weighted images were not included in MRI ccLS algorithm version 1.0 [19], since it was not inconsistently present in multiple MRI examinations at that time. It was even sometimes not considered necessary when using MRI ccLS algorithm version 2.0 [22, 26]. Nevertheless, we believed that diffusion-weighted images should be assessed, as the restriction on it is an important feature to determine the malignancy of SRM. Moreover, there is no formal definition for the term “homogeneity” in the MRI ccLS algorithm [15], which potentially leads to low inter-rater agreement and uncertainty in its application [17]. However, it was used to grade down the ccLS rating of a T2-hypointense intensely enhancing mass [14], because a fat-poor angiomyolipoma is more likely to be homogeneous than a ccRCC [46]. This unwritten rule should be further discussed, and corresponding modifications must be data-driven and rigorously validated [16]. Another challenge is the limited direct correlation between the histological types identified by the MRI ccLS algorithm and tumor grade or clinical management. The MRI ccLS algorithm can be useful in identifying ccRCC and papillary RCC [17]. It is also a reliable diagnostic method for differential diagnosis between renal epithelioid angiomyolipoma and ccRCC [47] and between renal oncocytoma and ccRCC [48]. The algorithm can effectively distinguish between certain renal tumor types, but it does not directly inform on the aggressiveness or optimal treatment strategy for each case. This limitation necessitates careful consideration of the the role of these algorithms in clinical decision-making, especially for patients under active surveillance who may be at risk of developing metastatic disease [14, 15, 20]. To address these issues, ongoing research, and long-term follow-ups are essential to validate the clinical utility of ccLS algorithms and determine their impact on patient outcomes.

The CT ccLS algorithm provides an alternative for SRM [27–29]. In comparison to the more comprehensive MRI ccLS algorithm, the CT ccLS algorithm is simpler and relies on mass-to-cortex corticomedullary enhancement ratios and tumor heterogeneity. In a similar fashion to the MRI ccLS algorithm, it is a five-tiered likelihood score system that gives the likelihood of ccRCC but does not aim to predict the histological subtype of SRM. The CT ccLS has the advantages of shorter examination times, lower cost, and increased accessibility. However, our study indicated that the sensitivity, specificity, and diagnostic odds ratio of MRI ccLS were higher than those of CT ccLS. Further, the region of confidence and predictive region of diagnostic performance was quite large for CT ccLS, indicating the limited robustness of the results. So far, there has been no head-to-head study comparing the diagnostic performance and cost-effectiveness of the MRI and CT ccLS algorithms in the diagnosis of ccRCC. Their complementary roles in SRM detection and surveillance have not yet been determined [29]. As there are more CT scans performed than MRI scans, more SRMs would be identified using CT. Therefore, the CT ccLS may be more frequently used in clinical practice. However, the MRI may still be necessary if the histology of SRM is warranted. It is also necessary to answer which procedure is more appropriate to be used as a modality for follow-up imaging of SRM in clinical practice. The future studies may focus on assessing the cost-effectiveness of these two algorithms to guide optimal clinical decision-making.

Our study has several limitations. First, most of the included studies are retrospective designed within a single center with a small sample size. As we have identified the risk of bias related to the index test and flow and timing, we encourage further validated the ccLS algorithms in prospective large-scale multiple-center studies to provide more robust evidence. Second, we did not pool the intra- and inter-reader agreement of the ccLS rating within and between radiologists with varied experience. It remains unknown whether the ccLS algorithms can work well if it is rated by inexperienced radiologists. Third, the quality assessment was based on specified signal questions. It is unclear whether these questions modified by our group can identify all the underlying sources of risk of bias assessment and applicability concerns. Fourth, we may exclude some of the eligible data due to the undeterminable risk of sample overlapping. However, we included the studies with the largest sample to make our results to be as close to the truth as possible. Fifth, we did not quantitively evaluate the source of heterogeneity in the meta-analysis of MRI ccLS due to the limited number of studies and underreported details. We supposed that the following factors could potentially introduce heterogeneity in these studies, including various MRI and CT protocols, heterogenous inclusion and exclusion criteria, different experiences of readers, two versions of ccLS MRI algorithms, etc. We plan to identify whether these factors are sources of heterogeneity when a sufficient number of studies with details are available. Finally, only a limited number of studies were included in our review. It resulted in a weak level of evidence rating for adopting MRI and CT ccLS algorithms due to insufficient cases. Therefore, the results of our meta-analysis should be interpreted with caution. However, we believe that future studies are not likely to provide opposing conclusions. Therefore, the level of evidence might be improved in the future.

In conclusion, both MRI and CT ccLS algorithms demonstrate promising diagnostic performance in identifying ccRCC among SRM. The clinical adoption of these algorithms is hindered by several factors, including unstandardized imaging protocol, nonadherence to the current guideline, and lacking in appropriate management suggestion. Nonetheless, the application of MRI and CT ccLS algorithms can lead to a more accurate and clinically useful characterization of SRM, ultimately providing patients with optimized decision-making options for initial management.

## Supplementary information

ELECTRONIC SUPPLEMENTARY MATERIAL

## Data Availability

All data generated or analyzed during this study are included in this published article and its additional files.

## Abbreviations

ccLS: Clear cell likelihood score
ccRCC: Clear cell renal cell carcinoma
CI: Confidence interval
HSROC: Hierarchical summary receiver operating characteristic
QUADAS-2: Modified quality assessment of diagnostic accuracy studies
SRM: Small renal mass
