# Supplementary material for: Is there enough evidence supporting the clinical adoption of clear cell likelihood score (ccLS)? An updated systematic review and meta-analysis
Source: Insights Imaging. 2024 Oct 9;15:242. doi: 10.1186/s13244-024-01829-y (PMC11464715; doi:10.1186/s13244-024-01829-y)
Supplement: Supplementary file 1 — ELECTRONIC SUPPLEMENTARY MATERIAL [file 13244_2024_1829_MOESM1_ESM.pdf]

Is there enough evidence supporting the clinical adoption of clear cell likelihood score (ccLS)? An updated systematic review and meta-analysis
ELECTRONIC SUPPLEMENTARY MATERIAL

List of Supplementary Material

Supplementary Note S1 Study protocol
Supplementary Note S2 Search strategy and study selection
Supplementary Note S3 Data extraction and quality assessment
Supplementary Note S4 Data synthesis and analysis

Supplementary Table S1 Data extraction tool
Supplementary Table S2 QUADAS-2 tool with modified questions to the study
Supplementary Table S3 Category of five levels of evidence based on meta-analyzes
Supplementary Table S4 Methodological aspect of included studies
Supplementary Table S5 Patient characteristics of included studies
Supplementary Table S6 Mass characteristics of included studies
Supplementary Table S7 Imaging protocol of included studies
Supplementary Table S8 Rating process of included studies
Supplementary Table S9 QUADAS-2 assessment by two reviewers and consensus results
Supplementary Table S10 Two-by-two data for meta-analysis
Supplementary Table S11 Leave-one-out sensitivity analysis

Supplementary Figure S1 MRI clear cell likelihood algorithm version 2.0
Supplementary Figure S2 CT clear cell likelihood algorithm

Supplementary Note S1 Study protocol

This systematic review has been registered via the International prospective register of systematic reviews (PROSPERO; https://www.crd.york.ac.uk/prospero/). The following is the details of the registration.

First draft date: 20 Mar 2024
Last edit date: 25 Mar 2024
PROSPERO ID: 527042/ CRD42024527042

Review title
A systematic review and meta-analysis of clear cell likelihood score (ccLS)

Review question
The diagnostic performance of clear cell likelihood score (ccLS) in clinical practice.

Searches
Our search will include the following electronic databases: PubMed, Embase, Web of Science, China National Knowledge Infrastructure, and Wanfang Data. The study should be published after 2017, since the ccLS system was proposed on 2017. The study should be published in English, Chinese, Japanese, German, or French, to allow detailed assessment by our group. The formal search string will be developed by a radiologist with experience in systematic review.

Condition or domain being studied
Small solid renal masses (SRMs) are a frequent incidental cross-sectional imaging finding. Up to 80% of SRMs are malignant with clear cell renal cell carcinoma (ccRCC) being the most common subtype of RCC, while Some subtypes of RCC, such as papillary RCC (pRCC) or chromophobe RCC (chrRCC), often exhibit indolent behavior and may be safely surveilled for growth. The clear cell likelihood score (ccLS) system was devised in recent years to create a systematic approach for assessing the MRI features of SRMs and assigning a ccRCC likelihood score. It has reported that this scoring system performed well in clinical practice, and a previous systematic review has concluded that the ccLS had moderate to high accuracy for identifying ccRCC from other RCC subtypes and with a moderate inter-reader agreement. However, this systematic review has meta-analyzed overlapping cohorts; therefore, the results of this review is of doubt. Further, the ccLS has been updated in 2022, and a CT-based ccLS has also been proposed. It is unclear whether these systems are effective in the clinical practice. Therefore, our systematic review aims to (re-)assess the diagnostic performance of ccLS.

Participants/population
Participants' inclusion criteria:
1) patients with small renal mass;
2) patients had undergone at least one pre-treatment contrast-enhanced CT or MRI scan;
3) patients with clear cell likelihood score (ccLS) assessment;
4) patients with histological results of the small renal mass.

Participants' exclusion criteria:
1) not human patients, e. g. cell line, xenotransplant;
2) not small renal mass, e. g. cystic lesions, mass >= 7cm
3) without performed imaging procedure or clear cell likelihood score (ccLS) not assessed;
4) without histological results of the small renal mass.

Intervention(s), exposure(s)
Patients with small renal mass underwent at least one pre-treatment contrast-enhanced CT or MRI for clear cell likelihood score (ccLS) assessment.

Comparator(s)/control
Standard-of-care imaging.

Types of study to be included
Studies describing the diagnostic performance of clear cell likelihood score (ccLS) based on contrast-enhanced CT or MRI will be included in this review. Studies must be with full-text available and sufficient information for assessing the methodological quality, risk of bias and concern on application.

Study inclusion criteria:
1) studies are reported in English, Japanese, Chinese, German or French with institutional full-text availability;
2) the cohort consists of patients with histological results of small renal mass;

3) patients had undergone at least one pre-treatment contrast-enhanced CT or MRI for clear cell likelihood score (ccLS) assessment.

Study exclusion criteria:
1) duplicate studies;
2) reviews, technical reports, letters to editors, comments to published studies, conference proceedings, case reports, brief communications and articles with insufficient information for assessing the methodological quality;
3) studies are reported other than English, Japanese, Chinese, German or French;
4) not human, not small renal mass.

Main outcome(s)
The characteristics of included study will be summarized. The diagnostic performance of clear cell likelihood score (ccLS) will be summarized. The methodological quality, and risk of bias and concern on application, will be assessed.

Measures of effect
The diagnostic performance of clear cell likelihood score (ccLS) will be summarized. The risk of bias and concern on application of studies will be assessed by modified Quality Assessment of Diagnostic Accuracy Studies (QUADAS-2) tool.

Additional outcome(s)
If there is a sufficient number of studies, a meta-analysis may be performed to present the diagnostic performance of clear cell likelihood score (ccLS).

Measures of effect
Measures will include diagnostic odds ratio, sensitivity and specificity, positive likelihood ratio, negative likelihood ratio, and aera under the receiver operating characteristic curve.

Data extraction (selection and coding)
A data collection tool will be established based on similar reviews and then trialed on two randomly chosen studies, which fulfilled all the inclusion criteria. These shall be used to train reviewers to appropriately apply the data extraction tool. Two-by-two tables were directly extracted, if documented, or reconstructed based on available data, for the potential meta-analysis.

Risk of bias (quality) assessment
The risk of bias and concern on application of studies will be assessed by modified Quality Assessment of Diagnostic Accuracy Studies (QUADAS-2) tool.

Strategy for data synthesis
A narrative synthesis will be provided with information presented in the text and/or tables to summarize and explain the characteristics and findings of the included studies. If a sufficient number of studies, a meta-analysis may be performed to present the diagnostic performance of clear cell likelihood score (ccLS). The The Stata software will be used. The diagnostic odds ratio (DOR) and its corresponding 95% confidence interval (CI) will be quantitatively synthesized as the main effect using the random-effects model, and the corresponding p value will be calculated. Sensitivity, specificity, positive and negative likelihood ratio, and their 95% CIs will be also calculated. A hierarchical summary receiver operating characteristic (HSROC) curve will be plotted to show the diagnostic performance. For heterogeneity assessment, Cochran's Q test and the Higgins I2 test will be used. For publication bias assessment, the Deeks funnel plot will be constructed, and the Deeks funnel asymmetry test will be s performed. Egger's and Begg's tests will be also conducted. A two-tailed p value > 0.10 will be indicate a low publication bias. The trim and fill method will be employed to evaluate the robustness of meta-analyses.

Analysis of subgroups or subsets
If a sufficiently homogeneous subset of studies analyzed a single outcome parameter, e. g. MRI clear cell likelihood score (ccLS) version 1.0 versus 2.0, a meta-analysis of this subgroup may be attempted.

Type and method of review
Type of review: Diagnostic, Meta-analysis, Systematic review
Health area of the review: Cancer, Urological

Dissemination plans
We plan to present our results of systematic review via presentations on conferences and peer-reviewed journals.

Keywords
Clear cell likelihood score, Small renal mass, Clear cell renal cell carcinoma

Any additional information
Although we have not identified a similar review via PROSPERO, we have found a similar published review (Tian J, Teng F, Xu H, Zhang D, Chi Y, Zhang H. Systematic review and meta-analysis of multiparametric MRI clear cell likelihood scores for classification of small renal masses. Front Oncol. 2022 Oct 26;12:1004502. doi: 10.3389/fonc.2022.1004502. PMID: 36387185; PMCID: PMC9641245.). However, this review has meta-analyzed overlapping cohorts, so the results of this review is of doubt. We will perform a systematic review with careful assessment of the overlapping data, to provide a more scientifically robust result on this topic.

Funding sources/sponsors
This study has received funding by National Natural Science Foundation of China (82302183, 82271934), Yangfan Project of Science and Technology Commission of Shanghai Municipality (22YF1442400), Research Found of Health Commission of Changing District, Shanghai Municipality (2023QN01), Laboratory Open Fund of Key Technology and Materials in Minimally Invasive Spine Surgery (2024JZWC-ZDA03, 2024JZWC-YBA07), and Research Fund of Tongren Hospital, Shanghai Jiao Tong University School of Medicine (TRKYRC-XX202204, TRYJ2021JC06, TRYXJH18, TRYXJH28). They played no role in the study design, data collection or analysis, decision to publish, or manuscript preparation.

Conflicts of interest
None.

Acknowledgement
This study is supported by TRILOGY (TongRen Imaging Learn-to-each-Other Group of Youth), a group of young radiologists from Department of Imaging, Tongren Hospital, Shanghai Jiao Tong University School of Medicine, who are working, learning, and playing together.

Supplementary Note S2 Search strategy and study selection

1. Search strategy

We firstly performed a preliminary search to confirm the availability of the search string. One of the reviewers has experience in developing the search strings. The reviewer developed the search stirrings and validated their feasibility via PubMed. Then the search string was translated for literature search via Embase, Web of Science, China National Knowledge Infrastructure, and Wanfang Data. The search string was developed by combining the variations of the terms of small renal mass, etc., CT or MRI, and clear cell likelihood score, ccLS. Then, we conducted the formal search to identify potential available articles. The search string in a previous systematic review (Tian J, Teng F, Xu H, Zhang D, Chi Y, Zhang H. Systematic review and meta-analysis of multiparametric MRI clear cell likelihood scores for classification of small renal masses. Front Oncol. 2022 Oct 26;12:1004502. doi: 10.3389/fonc.2022.1004502. PMID: 36387185; PMCID: PMC9641245.) was also used as a reference.

The literature search and study selection were duplicated by two independent reviewers with 5 and 6 years of experience in CT and MRI interpretation. One of these two reviewers can read articles in English, Chinese, Japanese, German and French. The disagreements were resolved by consults with the review group composed of radiologists, a urologist, a nephrologist, a biostatistical expert, a biomedical engineering expert, a MR scientist, a MR technician, and a journal expert.

1.1 PubMed Search Strategy

Available via https://pubmed.ncbi.nlm.nih.gov
Preliminary search date: 21 Mar 2024
Articles retrieved: 82 (Filter of publication year 2017-2024 not applied)
Formal search date: 31 Mar 2024
Articles retrieved: 60 (Filter of publication year 2017-2024 applied)

Search string:
('magnetic resonance imaging'[Mesh] OR magnetic resonance imaging OR magnetic resonance OR MRI OR MR OR 'tomography, x-ray computed'[Mesh] OR computed tomography OR CT) AND ('clear cell likelihood score' OR 'ccLS')

1.2 Embase Search Strategy

Available via www.embase.com
Preliminary search date: 21 Mar 2024
Articles retrieved: 62 (Filter of publication year 2017-2024 not applied)
Formal search date: 31 Mar 2024
Articles retrieved: 49 (Filter of publication year 2017-2024 applied)

Search string:
('magnetic resonance imaging'/exp OR 'magnetic resonance imaging' OR 'magnetic resonance imaging':ti,ab,kw OR 'magnetic resonance':ti,ab,kw OR mr:ti,ab,kw OR mri:ti,ab,kw OR 'computed tomography'/exp OR 'computed tomography' OR 'computed tomography':ti,ab,kw OR ct:ti,ab,kw) AND ('clear cell likelihood score':ti,ab,kw OR 'ccLS':ti,ab,kw)

1.3 Web of Science Search Strategy

Available via apps.webofknowledge.com
Preliminary search date: 21 Mar 2024
Articles retrieved: 56 (Filter of publication year 2017-2024 not applied)
Formal search date: 31 Mar 2024
Articles retrieved: 45 (Filter of publication year 2017-2024 applied)

Search string:
(TS=(magnetic resonance imaging) OR TS=(magnetic resonance) OR TS=(MRI) OR TS=(MR) OR TS=(computed tomography) OR TS=(CT)) AND (TS=(clear cell likelihood score) OR TS=(ccLS))

1.4 China National Knowledge Infrastructure Search Strategy

Available via http://www.cnki.net
Preliminary search date: 21 Mar 2024
Articles retrieved: 53 (Filter of publication year 2017-2024 not applied)

Formal search date: 31 Mar 2024
Articles retrieved: 24 (Filter of publication year 2017-2024 applied)

Search string:

TKA="透明细胞癌可能性评分" OR TKA="ccLS"

English translation:
'clear cell likelihood score' OR 'ccLS'

1.5 Wanfang Data Search Strategy
Available via https://www.wanfangdata.com.cn
Preliminary search date: 21 Mar 2024
Articles retrieved: 38 (Filter of publication year 2017-2024 not applied)
Formal search date: 31 Mar 2024
Articles retrieved: 15 (Filter of publication year 2017-2024 not applied)

Search string:
主题:("透明细胞癌可能性评分") OR 主题:("ccLS")

English translation:
'clear cell likelihood score' OR 'ccLS'

This study search strategy has been tested in a pilot search to confirm its feasibility on 21 Mar 2024. The formal study search was performed on 31 Mar 2024.

2. Study selection
Two reviewers screened and selected studies independently. Contact with the authors was sought if the full-text version was not accessible otherwise. The reference lists of included studies and relevant reviews identified through the search were screened for additional, potentially eligible articles. These two reviewers have 5 and 6 years of experience in CT and MRI interpretation. One of these two reviewers can read articles in English, Chinese, Japanese, German and French. In case of disagreements, the review group composed of radiologists, a urologist, a nephrologist, a biostatistical expert, a biomedical engineering expert, a MR scientist, a MR technician, and a journal expert, was consulted.

2.1 Study for systematic review
Study inclusion criteria:
(1) studies are reported in English, Japanese, Chinese, German or French with institutional full-text availability;
(2) the cohort consists of patients with histological results of small renal mass;
(3) patients had undergone at least one pre-treatment contrast-enhanced CT or MRI for clear cell likelihood score (ccLS) assessment.

Study exclusion criteria:
(1) duplicate studies;
(2) reviews, technical reports, letters to editors, comments to published studies, conference proceedings, case reports, brief communications and articles with insufficient information for assessing the methodological quality;
(3) studies are reported other than English, Japanese, Chinese, German or French;
(4) not human, not small renal mass.

According to the criteria, we assessed the following 16 studies in full-text.; included 11 studies into systematic review, and excluded 5 studies with justifications.

(1) Included studies for systematic review
1. Canvasser NE, Kay FU, Xi Y, Pinho DF, Costa D, de Leon AD, Khatri G, Leyendecker JR, Yokoo T, Lay A, Kavoussi N, Koseoglu E, Cadeddu JA, Pedrosa I. Diagnostic Accuracy of Multiparametric Magnetic Resonance Imaging to Identify Clear Cell Renal Cell Carcinoma in cT1a Renal Masses. J Urol. 2017 Oct;198(4):780-786. doi: 10.1016/j.juro.2017.04.089. Epub 2017 Apr 28. PMID: 28457802; PMCID: PMC5972826.
2. Johnson BA, Kim S, Steinberg RL, de Leon AD, Pedrosa I, Cadeddu JA. Diagnostic performance of prospectively assigned clear cell Likelihood scores (ccLS) in small renal masses at multiparametric magnetic resonance imaging. Urol Oncol. 2019 Dec;37(12):941-946. doi: 10.1016/j.urolonc.2019.07.023. Epub 2019 Sep 17. PMID: 31540830; PMCID: PMC6934987.
3. Steinberg RL, Rasmussen RG, Johnson BA, Ghandour R, De Leon AD, Xi Y, Yokoo T, Kim S, Kapur P, Cadeddu JA, Pedrosa I. Prospective performance of clear cell likelihood scores (ccLS) in renal masses evaluated with multiparametric magnetic resonance imaging. Eur Radiol. 2021 Jan;31(1):314-324. doi: 10.1007/s00330-020-07093-0. Epub 2020 Aug 8. PMID: 32770377; PMCID: PMC7755825.
4. Dunn M, Linehan V, Clarke SE, Keough V, Nelson R, Costa AF. Diagnostic Performance and Interreader Agreement of the MRI Clear Cell Likelihood Score for Characterization of cT1a and cT1b Solid Renal Masses: An External Validation Study. AJR Am J Roentgenol. 2022 Nov;219(5):793-803. doi: 10.2214/AJR.22.27378. Epub 2022 Jun 1. PMID: 35642765.

5. Schieda N, Davenport MS, Silverman SG, Bagga B, Barkmeier D, Blank Z, Curci NE, Doshi AM, Downey RT, Edney E, Granader E, Gujrathi I, Hibbert RM, Hindman N, Walsh C, Ramsay T, Shinagare AB, Pedrosa I. Multicenter Evaluation of Multiparametric MRI Clear Cell Likelihood Scores in Solid Indeterminate Small Renal Masses. Radiology. 2022 Jun;303(3):590-599. doi: 10.1148/radiol.211680. Epub 2022 Mar 15. Erratum in: Radiology. 2023 Mar;306(3):e239001. PMID: 35289659; PMCID: PMC9794383.
6. Hao Y, Guo H, Wang H, Xu W, Cui M, Zhang X, Zhao J, Bai X, Liu B, Ye H. [Interobserver agreement and diagnostic performance assessment of clear cell likelihood score using T2WI with fat suppression technique]. Zhong Hua Fang She Xue Za Zhi,2023,57(5):528-534. Chinese. doi: 10.3760/cma.j.cn112149-20221105-00889.
7. Hao Y, Gao S, Zhang X, Cui M, Ding X, Wang H, Yang D, Ye H, Wang H. [Comparison of diagnostic performance of Clear Cell Likelihood Score v1.0 and v2.0 for clear renal cell carcinoma]. Nan Fang Yi Ke Da Xue Xue Bao. 2023 May 20;43(5):800-806. Chinese. doi: 10.12122/j.issn.1673-4254.2023.05.16. PMID: 37313822; PMCID: PMC10267243.
8. Ibrahim A, Pelsser V, Anidjar M, Kaitoukov Y, Camlioglu E, Moosavi B. Performance of clear cell likelihood scores in characterizing solid renal masses at multiparametric MRI: an external validation study. Abdom Radiol (NY). 2023 Mar;48(3):1033-1043. doi: 10.1007/s00261-023-03799-z. Epub 2023 Jan 13. PMID: 36639532.
9. Al Nasibi K, Pickovsky JS, Eldehimi F, Flood TA, Lavallee LT, Tsampalieros AK, Schieda N. Development of a Multiparametric Renal CT Algorithm for Diagnosis of Clear Cell Renal Cell Carcinoma Among Small (≤ 4 cm) Solid Renal Masses. AJR Am J Roentgenol. 2022 Nov;219(5):814-823. doi: 10.2214/AJR.22.27971. Epub 2022 Jun 29. PMID: 35766532.
10. Eldihimi F, Walsh C, Hibbert RM, Nasibi KA, Pickovsky JS, Schieda N. Evaluation of a multiparametric renal CT algorithm for diagnosis of clear-cell renal cell carcinoma among small (≤ 4 cm) solid renal masses. Eur Radiol. 2023 Nov 16. doi: 10.1007/s00330-023-10434-4. Epub ahead of print. PMID: 37968475.
11. Lemieux S, Shen L, Liang T, Lo E, Chu Y, Kamaya A, Tse JR. External Validation of a Five-Tiered CT Algorithm for the Diagnosis of Clear Cell Renal Cell Carcinoma: A Retrospective Five-Reader Study. AJR Am J Roentgenol. 2023 Sep;221(3):334-343. doi: 10.2214/AJR.23.29151. Epub 2023 May 10. PMID: 37162037.

(2) Excluded studies with justifications

1. Hao YW, Zhang Y, Guo HP, Xu W, Bai X, Zhao J, Ding XH, Gao S, Cui MQ, Liu BC, Ye HY, Wang HY. Differentiation between renal epithelioid angiomyolipoma and clear cell renal cell carcinoma using clear cell likelihood score. Abdom Radiol (NY). 2023 Dec;48(12):3714-3727. doi: 10.1007/s00261-023-04034-5. Epub 2023 Sep 25. PMID: 37747536. (Not small renal cell, and no content on small renal mass available)
2. Cui MQ, He B, Xu W, Hao YW, Ding XH, Wang S, Bai X, Liu BC, Ye HY, Wang HY. [Value of clear cell likelihood score in differentiation between renal oncocytoma and clear cell renal cell carcinoma]. Zhonghua Yi Xue Za Zhi. 2022 Dec 20;102(47):3779-3785. Chinese. doi: 10.3760/cma.j.cn112137-20221020-02193. PMID: 36517429. (Not small renal cell, and no content on small renal mass available)
3. Rasmussen RG, Xi Y, Sibley RC 3rd, Lee CJ, Cadeddu JA, Pedrosa I. Association of Clear Cell Likelihood Score on MRI and Growth Kinetics of Small Solid Renal Masses on Active Surveillance. AJR Am J Roentgenol. 2022 Jan;218(1):101-110. doi: 10.2214/AJR.21.25979. Epub 2021 Jul 21. PMID: 34286596; PMCID: PMC8725913. (Not diagnostic accuracy assessment on ccLS)
4. Vazquez LC, Xi Y, Rasmussen RG, Venzor JER, Kapur P, Zhong H, Dai JC, Morgan TN, Cadeddu JA, Pedrosa I. Characterization of Demographical Histologic Diversity in Small Renal Masses With the Clear Cell Likelihood Score. J Comput Assist Tomogr. 2024 Jan 9. doi: 10.1097/RCT.0000000000001567. Epub ahead of print. PMID: 38213063. (Not diagnostic accuracy assessment on ccLS)
5. Wang XJ, Qu BQ, Zhou JP, Zhou QM, Lu YF, Pan Y, Xu JX, Miu YY, Wang HQ, Yu RS. A Non-Invasive Scoring System to Differential Diagnosis of Clear Cell Renal Cell Carcinoma (ccRCC) From Renal Angiomyolipoma Without Visible Fat (RAML-wvf) Based on CT Features. Front Oncol. 2021 Apr 23;11:633034. doi: 10.3389/fonc.2021.633034. PMID: 33968732; PMCID: PMC8103199. (Not ccLS)

(3) Extra study identified by browsing the reference lists of included studies and relevant reviews

The browsing of the reference lists of included studies did not detect extra potentially available study. We identified one study (Morgan T, Dai J, Kommidi V, Kusin S, Pedrosa I, Cadeddu J. Mp49- 02 clear cell likelihood scores (ccls) on multiparametric mri decreases benign pathology rates in patients with chronic kidney disease (ckd) being considered for extirpative nephron sparing surgery (nss). J Urol (2021) 206:e874–5. doi: 10.1097/JU.0000000000002075.02) by browsing the reference list a relevant review (Tian J, Teng F, Xu H, Zhang D, Chi Y, Zhang H. Systematic review and meta-analysis of multiparametric MRI clear cell likelihood scores for classification of small renal masses. Front Oncol. 2022 Oct 26;12:1004502. doi: 10.3389/fonc.2022.1004502. PMID: 36387185; PMCID: PMC9641245.). However, this study is a conference abstract. Therefore, this study is excluded.

2.2 Study for meta-analysis

As predetermined in the review protocol, if there is a sufficient number of studies, a meta-analysis may be performed to present the diagnostic performance of clear cell likelihood score (ccLS). Therefore, the meta-analysis on following questions is conducted: (1) the diagnostic performance of MRI ccLS; (2) the diagnostic performance of CT ccLS.

Study inclusion criteria:

(1) studies attempt to answer a similar question.
(2) studies sufficient data to extract the data for meta-analysis, or with those could be calculated using published data.
(3) the studies using largest cohort with histological results, when there were studies using partly or potentially overlapping cohort.

Study exclusion criteria:

(1) insufficient data and unavailable to be calculated after contact to authors.
(2) totally overlapping cohorts.
(3) the property of overlapping cohort cannot be excluded.

According to the criteria, we assessed the following 11 studies in detail.; included 9 studies into meta-analysis, and excluded 2 studies with justifications. The details of overlapping assessment were available in Supplementary Note S4.

(1) Included studies for meta-analysis

1. Canvasser NE, Kay FU, Xi Y, Pinho DF, Costa D, de Leon AD, Khatri G, Leyendecker JR, Yokoo T, Lay A, Kavoussi N, Koseoglu E, Cadeddu JA, Pedrosa I. Diagnostic Accuracy of Multiparametric Magnetic Resonance Imaging to Identify Clear Cell Renal Cell Carcinoma in cT1a Renal Masses. J Urol. 2017 Oct;198(4):780-786. doi: 10.1016/j.juro.2017.04.089. Epub 2017 Apr 28. PMID: 28457802; PMCID: PMC5972826.
2. Steinberg RL, Rasmussen RG, Johnson BA, Ghandour R, De Leon AD, Xi Y, Yokoo T, Kim S, Kapur P, Cadeddu JA, Pedrosa I. Prospective performance of clear cell likelihood scores (ccLS) in renal masses evaluated with multiparametric magnetic resonance imaging. Eur Radiol. 2021 Jan;31(1):314-324. doi: 10.1007/s00330-020-07093-0. Epub 2020 Aug 8. PMID: 32770377; PMCID: PMC7755825.
3. Dunn M, Linehan V, Clarke SE, Keough V, Nelson R, Costa AF. Diagnostic Performance and Interreader Agreement of the MRI Clear Cell Likelihood Score for Characterization of cT1a and cT1b Solid Renal Masses: An External Validation Study. AJR Am J Roentgenol. 2022 Nov;219(5):793-803. doi: 10.2214/AJR.22.27378. Epub 2022 Jun 1. PMID: 35642765.
4. Schieda N, Davenport MS, Silverman SG, Bagga B, Barkmeier D, Blank Z, Curci NE, Doshi AM, Downey RT, Edney E, Granader E, Gujrathi I, Hibbert RM, Hindman N, Walsh C, Ramsay T, Shinagare AB, Pedrosa I. Multicenter Evaluation of Multiparametric MRI Clear Cell Likelihood Scores in Solid Indeterminate Small Renal Masses. Radiology. 2022 Jun;303(3):590-599. doi: 10.1148/radiol.211680. Epub 2022 Mar 15. Erratum in: Radiology. 2023 Mar;306(3):e239001. PMID: 35289659; PMCID: PMC9794383.
5. Hao Y, Gao S, Zhang X, Cui M, Ding X, Wang H, Yang D, Ye H, Wang H. [Comparison of diagnostic performance of Clear Cell Likelihood Score v1.0 and v2.0 for clear renal cell carcinoma]. Nan Fang Yi Ke Da Xue Xue Bao. 2023 May 20;43(5):800-806. Chinese. doi: 10.12122/j.issn.1673-4254.2023.05.16. PMID: 37313822; PMCID: PMC10267243.
6. Ibrahim A, Pelsser V, Anidjar M, Kaitoukov Y, Camlioglu E, Moosavi B. Performance of clear cell likelihood scores in characterizing solid renal masses at multiparametric MRI: an external validation study. Abdom Radiol (NY). 2023 Mar;48(3):1033-1043. doi: 10.1007/s00261-023-03799-z. Epub 2023 Jan 13. PMID: 36639532.
7. Al Nasibi K, Pickovsky JS, Eldehimi F, Flood TA, Lavallee LT, Tsampalieros AK, Schieda N. Development of a Multiparametric Renal CT Algorithm for Diagnosis of Clear Cell Renal Cell Carcinoma Among Small (≤ 4 cm) Solid Renal Masses. AJR Am J Roentgenol. 2022 Nov;219(5):814-823. doi: 10.2214/AJR.22.27971. Epub 2022 Jun 29. PMID: 35766532.
8. Eldihimi F, Walsh C, Hibbert RM, Nasibi KA, Pickovsky JS, Schieda N. Evaluation of a multiparametric renal CT algorithm for diagnosis of clear-cell renal cell carcinoma among small (≤ 4 cm) solid renal masses. Eur Radiol. 2023 Nov 16. doi: 10.1007/s00330-023-10434-4. Epub ahead of print. PMID: 37968475.
9. Lemieux S, Shen L, Liang T, Lo E, Chu Y, Kamaya A, Tse JR. External Validation of a Five-Tiered CT Algorithm for the Diagnosis of Clear Cell Renal Cell Carcinoma: A Retrospective Five-Reader Study. AJR Am J Roentgenol. 2023 Sep;221(3):334-343. doi: 10.2214/AJR.23.29151. Epub 2023 May 10. PMID: 37162037.

(2) Excluded studies with justifications

1. Johnson BA, Kim S, Steinberg RL, de Leon AD, Pedrosa I, Cadeddu JA. Diagnostic performance of prospectively assigned clear cell Likelihood scores (ccLS) in small renal masses at multiparametric magnetic resonance imaging. Urol Oncol. 2019 Dec;37(12):941-946. doi: 10.1016/j.urolonc.2019.07.023. Epub 2019 Sep 17. PMID: 31540830; PMCID: PMC6934987. (Overlapping cohort)
2. Hao Y, Guo H, Wang H, Xu W, Cui M, Zhang X, Zhao J, Bai X, Liu B, Ye H. [Interobserver agreement and diagnostic performance assessment of clear cell likelihood score using T2WI with fat suppression technique]. Zhong Hua Fang She Xue Za Zhi,2023,57(5):528-534. Chinese. doi: 10.3760/cma.j.cn112149-20221105-00889. (Overlapping cohort)

Supplementary Note S3 Data extraction and quality assessment

We developed a data extraction sheet to collect study data. As the reviewers have different levels of experience and knowledge, the items listed were reviewed and discussed to ensure that all reviewers had clear knowledge of the procedures. A training phase was introduced before the formal extraction. During the training phase, two randomly chosen articles from all articles fulfilled the inclusion criteria for discussion were used to train reviewers. They thoroughly read the two randomly chosen articles including the supplementary materials, and measured each study independently. A structured data collection tool was modified and used to help them reach agreement. Disagreements were discussed in order to achieve a shared understanding of each parameter. This pre-defined and piloted data extraction tool was used in the formal data extraction phase. The details of the data extraction tool can be found in Supplementary Table S1. In addition to the basic information of the study, the two-by-two data for meta-analysis were extracted with caution of the overlapping cohorts. The details for the extraction and reconstruction of two-by-two data were described in Supplementary Note S4.

The data extraction was duplicated by two independent reviewers with 5 and 6 years of experience in CT and MRI interpretation. One of these two reviewers can read articles in English, Chinese, Japanese, German and French. The disagreements were resolved by consults with the review group composed of radiologists, a urologist, a nephrologist, a biostatistical expert, a biomedical engineering expert, a MR scientist, a MR technician, and a journal expert.

The following items has been discussed:
(1) Methodological aspect - Study design
The definition of a retrospective study is clear that retrospectively collect the data and rated ccLS for the renal masses. The retrospective analysis of prospective rated ccLS is considered as a prospective study in our review. This kind of study prospective rated the ccLS as a clinical routine of structured report for renal masses.
(2) Methodological aspect - ccLS algorithm
The ccLS algorithm includes MRI ccLS algorithm and CT ccLS algorithm. It is clear whether an MRI ccLS algorithm or a CT ccLS algorithm was used. However, it may be hard to determine whether an MRI ccLS algorithm version 1.0 or 2.0 was used. We determined the version of MRI ccLS algorithm according to the declaration of "version 1.0 or 2.0" in the manuscript, the citation of the ccLS in the manuscript, and by comparing the year of receive and the publish of MRI ccLS algorithm version 1.0 and 2.0.
(3) Diagnostic performance – cutoff for ccLS
The cutoff of ccLS for identifying ccRCC should be >= 4 in our review. Although we found that some the studies may analyze the data using multiple cutoffs, we would only extract the data using the cutoff of >= 4 for ccRCC.
(4) Diagnostic performance - Number of Events (True Positive, False Positive, False Negative, True Negative)
The two-by-two table for each study was directly extracted if reported in the study, or reconstructed with all the available data in the manuscript. The size of tumor should be <= 4 cm in our review. Although we found that some the studies may analyze the data of tumors > 4 cm, we would only extract the data using the tumor with size <= 4 cm.
(5) Diagnostic performance - Sensitivity, Specificity, Positive Predictive Value (PPV), Negative Predictive Value (NPV), Accuracy
These diagnostic performance metrics were extracted for reconstruction of the Number of Events (True Positive, False Positive, False Negative, True Negative) data. If there was an overall diagnostic performance metrics data for the study, we would use the overall data to present the overall diagnostic performance of ccLS algorithm by all the raters in the study. However, when overall data was not available, we would use the data form individual rater with the lowest aera of curve or accuracy, to allow conservative estimates for the diagnostic performance of the ccLS algorithm.

2. Risk of bias assessment
The risk of bias assessment was performed using the modified Quality Assessment of Diagnostic Accuracy Studies (QUADAS-2) tool. As the reviewers have different levels of experience and knowledge, the items listed were reviewed and discussed to ensure that all reviewers had clear knowledge of the procedures. A training phase was introduced before the formal assessment. During the training phase, two randomly chosen articles from all articles fulfilled the inclusion criteria for discussion were used to train reviewers. They thoroughly read the two randomly chosen articles including the supplementary materials, and discussed the items in QUADAS-2 tool to reach agreement. The details of the tool can be found in Supplementary Table S2.

The risk of bias assessment was duplicated by two independent reviewers with 5 and 6 years of experience in CT and MRI interpretation. One of these two reviewers can read articles in English, Chinese, Japanese, German and French. The disagreements were resolved by consults with the review group composed of radiologists, a urologist, a nephrologist, a biostatistical expert, a biomedical engineering expert, a MR scientist, a MR technician, and a journal expert.

The following items has been discussed:
(1) Index test - Signaling question 1: were the imaging protocol described in detail?
We discussed how to define a detailed imaging acquisition protocol. We believed that it is not necessary to provide all

the imaging parameters for such an image rating system, since it is rated by naked-eye of radiologists. The radiologists can adopt to images with diverse scanners and imaging parameters. Therefore, we define a detailed imaging acquisition protocol as a protocol with all the MRI sequence names for assessment, or all the phase of CT scan for assessment. It is not necessary or possible to force all the institutions that attempt to use the ccLS using the same imaging acquisition protocol, but comparable ones.
(2) Index test - Signaling question 2: were the rater trained for ccLS before formal rating?
We believe it is necessary to describe the training session of ccLS before the formal assessment. The ccLS was not a routine content in all the resident or abdominal fellowship training. It is necessary to pre-train the raters before the ccLS assessment studies, so that they can use the system properly to present the true diagnostic performance of the system.
(3) Index test - Signaling question 3: was the rating process of ccLS approach described in detail (no. of rater, experience, blindness, consensus method)?
We believe it is necessary to describe the rating process of ccLS approach in detail, so that the future studies can repeat the study to confirm the results. It is also important to describe the rating process in detail to allow clinical adoption of ccLS as a routine in small renal mass assessment. Ideally, the the rating process of ccLS approach should mimic the clinical reporting process; however, it may be difficult in a “experimental” diagnostic accuracy study.
(4) Reference - Signaling question 1: was the reference standard adequate (histological evidence from biopsy or surgery)?
All the results of the small renal mass diagnosis should have histological evidence. However, the source of the histological evidence includes surgery and biopsy. It may be not a clinical routine for a mass with low ccLS rating to undergo surgery; therefore, we also accept histological evidence from biopsy. However, in comparison to the histological evidence from surgery, it may introduce to some extent risk of bias.
(5) Flow and timing - Signaling question 1: was there an appropriate interval (an interval less than 3 months) between index test and reference standard?
The key of the flow and timing in a diagnostic accuracy study is the interval between the index test and reference standard, i. e., the interval between the imaging and the surgery or biopsy. Not all the studies reported the interval between the imaging and the surgery or biopsy. We considered that these studies should be rated with unclear risk of bias since the interval is unknown. In studies that reported the interval between the imaging and the surgery or biopsy, the key is how to decide whether the interval is adequate. As the follow-up for a renal mass or surgery would be 3, 6, 12, 24… months, which indicated that the mass may change within 3 months. We chose the cutoff of 3 month for the low and high risk of flow and timing assessment.

Supplementary Note S4 Data synthesis and analysis

1. Statistical analysis

A narrative synthesis will be provided with information presented in the text and/or tables to summarize and explain the characteristics and findings of the included studies. The QUADAS-2 assessment was qualitatively summarized. The sample size, number of ccRCC patients, and non-ccRCC patients, and other histological subtype of renal mass, were quantitatively summarized. The rate (events/sample size) of ccRCC patients, and non-ccRCC patients, and other histological subtype of renal mass, were treated as a ratio in pooled analysis. The statistical analysis was performed using SPSS software version 26.0. A two-tailed alpha level of 0.05 was set, unless otherwise specified.

2. Handling overlapping study

Our systematic review included 11 studies, the overlapping data was carefully identified by comparing the register number, study institution, and inclusion period. As described in Supplementary Note S2, we only included the studies using largest cohort with histological results, when there were studies using partly or potentially overlapping cohort. The studies were excluded when the cohorts were totally overlapping or there was potential of overlapping cohort. The following presents the study included for meta-analysis.

The following studies were assessed.

(1) Three studies from University of Texas Southwestern Medical Center

Canvasser2017 (University of Texas Southwestern Medical Center, 2011.12 to 2015.07); Johnson2019 (University of Texas Southwestern Medical Center, 2016.06 to 2018.04); Steinberg2021 (University of Texas Southwestern Medical Center; Parkland Health and Hospital System, 2016.06 to 2019.11). The inclusion period of two studies were overlapping (Johnson2019 and Steinberg2021), we included the one with longer inclusion period (Steinberg2021), and excluded the one with shorter inclusion period (Johnson2019).

(2) Two studies from First Medical Center, Chinese PLA General Hospital

Hao2023A (First Medical Center, Chinese PLA General Hospital, 2021.01 to 2021.12); Hao2023B (First Medical Center of Chinese PLA General Hospital, 2018.01 to 2021.12; Beijing Friendship Hospital, Capital Medical University; Peking University First Hospital, 2019.01 to 2021.05). The inclusion period of these two studies were overlapping, we included the one with longer inclusion period and more institution (Hao2023B), and excluded the one with shorter inclusion period (Hao2023A).

(3) Two studies from The Ottawa Hospital

Nasibi2022 (The Ottawa Hospital, 2016.01 to 2019.12); Eldihimi2023 (The Ottawa Hospital, 2014.10 to 2021.01). Although the inclusion period of these two studies were overlapping, they were treated as different cohorts. First, they patients of these two studies were patients who underwent surgery or biopsy, respectively. Second, the study subjects or cohorts overlap statement of the Eldihimi2023 declared that none of the study subjects or cohorts were overlapping.

3. Meta-analysis

The meta-analysis was conducted using R language within RStudio with relevant packages and their corresponding website applications, namely MetaDTA (https://crsu.shinyapps.io/MetaDTA/), and metaumbrella (https://www.metaumbrella.org). These two website applications are handy tools for reviewers to perform diagnostic test accuracy meta-analysis. If a sufficient number of studies, a meta-analysis may be performed to present the diagnostic performance of clear cell likelihood score (ccLS). In current study, the meta-analysis on following questions was conducted: (1) the diagnostic performance of MRI ccLS; (2) the diagnostic performance of CT ccLS.

The diagnostic odds ratio (DOR) and its corresponding 95% confidence interval (CI) was quantitatively synthesized as the main effect using the random-effects model, and the corresponding p value was calculated. Since there is high heterogeneity among different studies in study design, patient characteristics, MRI or CT protocols, the random model was selected. Sensitivity, specificity, positive and negative likelihood ratio, and their 95% CIs were also calculated.

We did not perform a subgroup analysis or meta-regression for investigating the heterogeneity, because there was not a sufficiently homogeneous subset of studies with detailed reporting. However, the reliability of the pooled effect size was assessed, by a sensitivity analysis omitting the included studies one by one. A hierarchical summary receiver operating characteristic (HSROC) curve was plotted to show the diagnostic performance.

For heterogeneity assessment, we selected the Higgins I2 test rather than Cochran's Q test. Measuring inter-study dispersion assumes that, if all studies were methodologically identical and variation in results were only due to the random selection of study participants, the effect sizes would follow a chi-squared distribution. Cochran's Q assesses the hypothesis that the distribution of results is homogenous and p-values < 0.05 would generally lead to the rejection of this null-hypothesis. As with a small number of studies Cochran's Q can be distorted, I2, a measure for how much of the variability between effect size estimates is due to methodological heterogeneity rather than sampling error, was selected in our study. I2 values of 25% and less are usually considered to be low or unimportant, 25% to 50% moderate and values above 75% are considered high.

The 95% prediction intervals (PI) were calculated to facilitate more conservative prediction for potential application of ccLS. The Egger's test was conducted for small-study effects and publication bias. A two-tailed p value > 0.10 was indicate a low publication bias. Excess significance bias was evaluated by a Chi-square test comparing the actual observed number of primary studies with a p value < 0.05 with the expected number of primary studies with statistical significance.

4. Level of evidence rating

The strength of evidence supporting radiomics for clinical use were categorized into five levels: convincing, highly suggestive, suggestive, weak, and not suggestive (Supplementary Table S3). The rating was based on the results of a series of aforementioned analyses. This function is available using a website metaumbrella application (https://www.metaumbrella.org). This website application is an easy-to-use tool for reviewers to perform evidence level rating according to the results of meta-analyses.

The criteria were strongly recommended to be used to all allow an objective, standardized classification of the level of evidence. However, the analysts should not forget that the variables used in these criteria are continuous and the set of cut-off points are only cut-off points. For example, the difference between a factor that includes 1000 patients and a factor that includes 1001 patients is negligible, but according to the criteria, the former can only be class IV (weak), whereas the latter could be class I (convincing).

Supplementary Table S1 Data extraction tool

Field	Item
Bibliographic information	The title of the study
	Published year
	Published journal
	Impact factor of published journal
	Published volume
	Published issue
	Published page
	Study ID, determined by First Author + Year, + A, B, C, if needed
Methodological aspect	Study design
	Study center
	Study period
	ccLS algorithm
	No. of Rater
	Interval between ccLS and reference
	Reference standard
Patient characteristics	No. of patients
	Age
	Gender
	Inclusion criteria
	Exclusion criteria
	No. of mass
	Tumor size
	Histologic diagnosis
Imaging protocol of included studies	Imaging modality
	Standard for protocol
	Scanner
	Imaging protocol
Rating process	No. of rater
	Experience of rater
	Training session
	Blindness
	Consensus method
Diagnostic performance	Number of Events (True Positive, False Positive, False Negative, True Negative)
	Cutoff for ccRCC
	Sensitivity
	Specificity
	Accuracy
	Positive Predictive Value (PPV)
	Negative Predictive Value (NPV)
	Positive Likelihood Ratio (PLR)
	Negative Likelihood Ratio (NLR)
	Diagnostic Odds Ratio (DOR)

Supplementary Table S2 QUADAS-2 tool with modified questions to the study

<table>
<tr><th>Domain and Description</th><th>Modified signaling question</th><th>Risk of bias</th><th>Applicability concern</th></tr>
<tr><td rowspan="3">Patient selection - describe methods of patient selection: Describe included patients (prior testing, presentation, intended use of index test and setting)</td><td>Signaling question 1: was the type of study (retrospective or prospective) specified?</td><td rowspan="3">Could the selection of patients have introduced bias?</td><td rowspan="3">Are there concerns that the included patients do not match the review question?</td></tr>
<tr><td>Signaling question 2: were the study institution and inclusion period clearly presented?</td></tr>
<tr><td>Signaling question 3: were the inclusion/exclusion criteria specified?</td></tr>
<tr><td rowspan="3">Index test - describe the index test and how it was conducted and interpreted</td><td>Signaling question 1: were the imaging protocol described in detail?</td><td rowspan="3">Could the conduct or interpretation of the index test have introduced bias?</td><td rowspan="3">Are there concerns that the index test, its conduct, or interpretation differ from the review question?</td></tr>
<tr><td>Signaling question 2: were the rater trained for ccLS before formal rating?</td></tr>
<tr><td>Signaling question 3: was the rating process of ccLS approach described in detail (no. of rater, experience, blindness, consensus method)?</td></tr>
<tr><td rowspan="2">Reference standard - describe the reference standard and how it was conducted and interpreted</td><td>Signaling question 1: was the reference standard adequate (histological evidence from biopsy or surgery)?</td><td rowspan="2">Could the reference standard, its conduct, or its interpretation have introduced bias?</td><td rowspan="2">Are there concerns that the target condition as defined by the reference standard does not match the review question?</td></tr>
<tr><td>Signaling question 2: was the reference standard established by an experienced pathologist with an adequate cirteria?</td></tr>
<tr><td>Flow and timing - describe any patients who did not receive the index test(s) and/or reference standard or who were excluded from the 2x2 table (refer to flow diagram): Describe the time interval and any interventions between index test(s) and reference standard</td><td>Signaling question 1: was there an appropriate interval (an interval less than 3 months) between index test and reference standard?</td><td>Could the patient flow have introduced bias?</td><td>n. a.</td></tr>
</table>

Note: Whiting PF, Rutjes AW, Westwood ME, Mallett S, Deeks JJ, Reitsma JB, Leeflang MM, Sterne JA, Bossuyt PM; QUADAS-2 Group. QUADAS-2: a revised tool for the quality assessment of diagnostic accuracy studies. Ann Intern Med. 2011 Oct 18;155(8):529-36. doi: 10.7326/0003-4819-155-8-201110180-00009. PMID: 22007046.

Supplementary Table S3 Category of five levels of evidence based on meta-analyzes

**Levels of supporting evidence**	**Description**
Convincing	$p < 10^{-6}$, > 1000 events, the largest study reaches statistical significance ($p < 0.05$), I2 < 50%, the null value excluded by the 95% PI, no small-study effects ($p > 0.1$ for Egger's test) and excess significance ($p > 0.1$).
Highly suggestive	$p < 10^{-6}$, > 1000 events, the largest study reaches statistical significance ($p < 0.05$)
Suggestive	$p < 10^{-3}$, > 1000 events
Weak	$p < 0.05$
Not suggestive	$p > 0.05$

Note: Fusar-Poli P, Radua J. Ten simple rules for conducting umbrella reviews. Evid Based Ment Health. 2018 Aug;21(3):95-100. doi: 10.1136/ebmental-2018-300014. Epub 2018 Jul 13. PMID: 30006442.

Supplementary Table S4 Methodological aspect of included studies

Study [Reference]	Author	Year	Journal	Study design	Study center	Study period	ccLS algorithm	No. of Rater	Interval between ccLS and reference	Reference standard
Canvasser2017 [19]	Canvasser	2017	J Urol	Retrospective	University of Texas Southwestern Medical Center	2011.12 to 2015.07	MRI ccLS v1.0	1 of 7	Not reported	Histology after surgery
Johnson2019 [20]	Johnson	2019	Urol Oncol	Prospective	University of Texas Southwestern Medical Center	2016.06 to 2018.04	MRI ccLS v1.0	1 of 14	Not reported	Histology after surgery or biopsy
Steinberg2021 [22]	Steinberg	2021	Eur Radiol	Prospective	University of Texas Southwestern Medical Center; Parkland Health and Hospital System	2016.06 to 2019.11	MRI ccLS v1.0	1 of 16	Not reported	Histology after surgery or biopsy
Dunn2022 [23]	Dunn	2022	AJR Am J Roentgenol	Retrospective	Queen Elizabeth II Health Sciences Centre and Dalhousie University	2013.01 to 2018.02	MRI ccLS v2.0	3	Not reported	Histology after surgery or biopsy
Schieda2022 [24]	Schieda	2022	Radiology	Retrospective	Ottawa Health Science Network Research Ethics Board; University of Nebraska Medical Center; Mass General Brigham; Michigan Medicine; NYU Grossman School of Medicine	2012.12 to 2019.12	MRI ccLS v2.0	2 of 10	< 12 month	Histology after surgery or biopsy
Hao2023A [25]	Hao	2023	Chinese Journal of Radiology	Retrospective	First Medical Center, Chinese PLA General Hospital	2021.01 to 2021.12	MRI ccLS v2.0	2	< 1 month	Histology after surgery or biopsy
Hao2023B [26]	Hao	2023	Journal of Southern Medical University	Retrospective	First Medical Center of Chinese PLA General Hospital; Beijing Friendship Hospital, Capital Medical University; Peking University First Hospital	2018.01 to 2021.12 for A; 2019.01 to 2021.05 for B and C	MRI ccLS v1.0 and v2.0	2 of 6	< 1 month	Histology after surgery or biopsy
Ibrahim2023 [27]	Ibrahim	2023	Abdom Radiol (NY)	Retrospective	Jewish General Hospital, McGill University	2021.01 to 202.12	MRI ccLS v2.0	2	Not reported	Histology after surgery or biopsy
AlNasibi2022	Al Nasibi	2022	AJR Am J	Retrospective	The Ottawa Hospital	2016.01 to	CT ccLS	2	<12 month	Histology

[28]			Roentgenol			2019.12				after surgery
Eldihimi2023 [29]	Eldihimi	2023	Eur Radiol	Retrospective	The Ottawa Hospital	2014.10 to 2021.01	CT ccLS	3	<12 month	Histology after biopsy
Lemieux2023 [30]	Lemieux	2023	AJR Am J Roentgenol	Retrospective	Stanford University School of Medicine	2012.01 to 2022.07	CT ccLS	5	<12 month	Histology after surgery

Note: ccLS = clear cell likelihood score.

Supplementary Table S5 Patient characteristics of included studies

Study [Reference]	No. of patient	Age	Gender (M/F)	Inclusion criteria	Exclusion criteria
Canvasser2017 [19]	110	57 ± 14	61/39	Patients with cT1a renal masses who underwent partial or radical nephrectomy and preoperative multiparametric MRI between December 2011 and July 2015	patients with poor quality or limited MRI examinations (i. e., not including the sequences described)
Johnson2019 [20]	57	67.1 ± 14.9	38/19	(1) patients who underwent a multiparametric MRI to evaluate a cT1a renal mass and received a ccLS from June 2016 until April 2018;<br>(2) had histological confirmation of the renal mass by renal biopsy and/or surgical resection	(1) unable to complete multiparametric MRI examination;<br>(2) nondiagnostic renal biopsy of the mass or no surgical pathology available
Steinberg2021 [22]	204	59 ± 13	110/94	Patients who underwent a multiparametric MRI to evaluate a solid renal mass during the study period with subsequent confirmatory histologic diagnosis	(1) unable to complete multiparametric MRI or multiparametric MRI performed without IV contrast;<br>(2) associated histopathology or biopsy prior to multiparametric MRI;<br>(3) masses excluded by the ccLS algorithm (e.g., presence of macroscopic fat, masses with less than 25% solid component)
Dunn2022 [23]	NR	56.5 ± 12.9	NR	Histologically confirmed renal neoplasms that were diagnosed be- tween January 2013 and February 2018	(1) tumor-related factors: size greater than 7 cm, size less than 1 cm, presence of local invasion or metastatic disease on histology or imaging, cystic renal mass (defined as < 25% enhancing tissue per the Bosniak cyst classification system version 2019), presence of macroscopic fat on imaging, prior intervention, and mixed or undifferentiated histology;<br>(2) MRI-related factors: severe artifacts, MRI protocol missing sequences required for application of the ccLS v2.0 algorithm, and no IV contrast material.
Schieda2022 [24]	241	60 ± 13	174/67	(1) Adult patients >=18 years old;<br>(2) CT1a <=4 cm solid (approximately ≥25% of the volume enhancing) renal mass;<br>(3) multiparametric MRI;<br>(4) Histological diagnosis within 1 year of MRI	(1) Infiltrative, non-circumscribed renal mass;<br>(2) macroscopic fat within renal mass on CT or MRI;<br>(3) Any patient with a known genetic syndrome predisposing to renal masses;<br>(4) Patients with more than 3 solid renal masses in a single kidney;<br>(5) Incomplete multi-parametric MRI protocol or MRI performed at an outside institution;<br>Histological diagnosis provided by outside

					pathologists or obtained from a procedure other than nephrectomy or 18-gauge or larger core needle biopsy
Hao2023A [25]	111	55 ± 12	77/34	(1) tumor size 1˜4 cm;<br>(2) renal mass with approximately ≥25% of the volume enhancing;<br>(3) complete MRI scan, including T2WI, FS-T2WI, DWI, in/opp, T1WI, and T1WI+C;<br>(4) complete clinical history;<br>(5) Histological diagnosis within 1 month of MRI	(1) Pre-treatment before MRI;<br>(2) poor image quality
Hao2023B [26]	691	54 ± 12	491/200	(1) tumor size 1˜4 cm;<br>(2) renal mass with approximately ≥25% of the volume enhancing;<br>(3) complete MRI scan, including T2WI, FS-T2WI, DWI, in/opp, T1WI, and T1WI+C;<br>(4) complete clinical history;<br>(5) Histological diagnosis within 1 month of MRI	(1) Pre-treatment before MRI;<br>(2) poor image quality
Ibrahim2023 [27]	59	63 ± 12	36/23	Adult patients with solid (≥ 25% of the enhancing volume) renal masses and histological diagnosis within 1 year after multiparametric MRI	(1) inadequate or incomplete multiparametric MRI examination;<br>(2) without histopathologic diagnosis;<br>(3) with infiltrating masses, masses containing macroscopic fat or cystic masses (> 75%)
AlNasibi2022 [28]	148	58 ± 12	73/75	Renal masses in adult patients (age ≥ 18 years) that were imaged by CT before surgical resection with a pathologic diagnosis.	(1) CT did not use a complete renal protocol;<br>(2) surgery was performed more than 12 months after CT;<br>(3) the appearance of the resected mass on CT was predominantly cystic (< 25% enhancing internal elements);<br>(4) there was macroscopic fat in the resected mass on CT;<br>(5) the size of the resected mass (based on mean of axial short-axis measurement, axial long-axis measurement, and coronal measurement) was greater than 4 cm on CT<br>(6) in patients with multiple masses on CT, a known or suspected hereditary renal cancer syndrome or more than three masses in a single kidney
Eldihimi2023 [29]	51	67 ± 12	33/18	Renal masses in adult patients (age≥18 years) that were imaged by CT prior to renal mass biopsy, yielding a pathologic diagnosis	(1) CT did not use a complete renal protocol or biopsy was performed>12 months after CT;<br>(2) size of resected mass (based on mean of axial short-axis measurement, axial long-axis

					measurement, and coronal measurement) greater than 4 cm on CT
Lemieux2023 [30]	93	62 (52-69)	51/45	Consecutive adult patients (18 years old or older) who underwent abdominal CT from January 2012 through July 2022 and for whom the report impression included the phrase "renal mass," "renal cell carcinoma," "RCC," "renal neoplasm," "renal cell neoplasm," or "kidney cancer" and who subsequently underwent either a partial nephrectomy or radical nephrectomy at Stanford University Medical Center	(1) review of electronic medical record: CT images were unavailable for review, no renal mass was identified in the pathology report (i.e., patient underwent en bloc kidney resection for a retroperitoneal malignancy), CT was performed more than 1 year before surgery, and the patient had known hereditary renal mass syndrome;<br>(2) first CT image review: availability of only an unenhanced phase, presence of more than three renal masses (excluding simple cysts) in a single kidney, and presence of nodal or metastatic disease<br>(3) second CT image review: macroscopic fat in mass (four masses), cystic mass (defined as presence of 25% or less enhancing components), and mass size greater than 4 cm (i.e., not a mass designated cT1a or lower);<br>(4) review by an attending radiologist: characteristics of the CT examinations for the potentially eligible masses<br>(5) subsequent review by an attending radiologist: No corticomedullary phase, motion artifact

Note: ccLS = clear cell likelihood score, NR = not reported, T1WI = T1 weighted images, T1WI + C= T1 weighted images with contrast enhancement, FS-T2WI = fat-saturated T2 weighted images, T2WI = T2 weighted images, DWI = diffusion weighted images.

Supplementary Table S6 Mass characteristics of included studies

Study [Reference]	No. of mass	Tumor size	No. of ccRCC	No. of papillary RCC	No. of chromophobe RCC	No. of other RCC	No. of oncocytic neoplasm	No. of oncocytoma	No. of angiomyolipoma	No. of benign lesions
Canvasser2017 [19]	121	2.4 ± 0.8	61	25	9	6	0	7	6	7
Johnson2019 [20]	63	2.7 ± 0.7	35	14	3	0	3	5	0	3
Steinberg2021 [22]	213	2.7 ± 0.8	120	44	8	15	2	14	0	10
Dunn2022 [23]	83	3.0 ± 1.3	34	22	9	0	0	12	6	0
Schieda2022 [24]	250	2.5 ± 0.8	119	57	14	12	6	27	10	4
Hao2023A [25]	111	2.8 ± 0.9	82	7	3	2	1	0	13	1
Hao2023B [26]	700	2.7 ± 1.7	509	50	40	0	0	17	49	0
Ibrahim2023 [27]	61	2.3 ± 0.9	25	9	2	2	0	14	1	8
AlNasibi2022 [28]	148	2.6 ± 0.6 for ccRCC; 2.2 ± 0.7 for non-ccRCC	78	22	9	4	6	19	7	3
Eldihimi2023 [29]	51	2.8 ± 0.8 for ccRCC; 2.5 ± 0.8 for non-ccRCC	26	8	3	2	1	9	2	0
Lemieux2023 [30]	97	2.4 (1.7-3.2)	43	9	5	2	2	21	11	4

Note: ccLS = clear cell likelihood score, ccRCC = clear cell renal cell carcinoma.

Supplementary Table S7 Imaging protocol of included studies

Study [Reference]	Imaging modality	Standard for protocol	Scanner	Imaging protocol
Canvasser2017 [19]	MRI	Diverse imaging protocols in multiple institutions	Diverse 1.5-T and 3.0-T scanners	Axial and coronal FS-T2WI; Axial chemical shift T1WI; Axial or Coronal FS-dynamic contrast enhanced T1WI (corticomedullary and late nephrographic, and/or excretory phases)
Johnson2019 [20]	MRI	Institutional standard clinical imaging protocol	Diverse 1.5-T and 3.0-T scanners	Axial and coronal FS-T2WI; Axial chemical shift T1WI; DWI with b = 0, 50, 400, 800 (ADC); Axial or Coronal FS-dynamic contrast enhanced T1WI (corticomedullary and late nephrographic, and excretory phases)
Steinberg2021 [22]	MRI	Previously described standard clinical MRI protocol	Diverse 1.5-T and 3.0-T scanners	Axial and coronal FS-T2WI; Axial chemical shift T1WI; DWI with b = 0, 50, 400, 800 (ADC); Axial or Coronal FS-dynamic contrast enhanced T1WI (corticomedullary, late nephrographic, and excretory phases)
Dunn2022 [23]	MRI	NR	Diverse 1.5-T scanners	Axial and coronal FS-T2WI; Axial chemical shift T1WI; Axial or Coronal FS-dynamic contrast enhanced T1WI (corticomedullary, and delay phases)
Schieda2022 [24]	MRI	Diverse imaging protocols in multiple institutions	Diverse 1.5-T and 3.0-T scanners	Axial and/or coronal FS-T2WI; FS-T2WI; Axial chemical shift T1WI; DWI with b <= 200, b >=500; Axial or Coronal FS-dynamic contrast enhanced T1WI (corticomedullary and late nephrographic phases)
Hao2023A [25]	MRI	Institutional standard clinical imaging protocol	One 3.0-T scanner	FS-T2WI and T2WI; chemical shift T1WI; DWI; FS-dynamic contrast enhanced T1WI (corticomedullary, late nephrographic, and excretory phases)
Hao2023B [26]	MRI	Diverse imaging protocols in multiple institutions	Diverse 1.5-T and 3.0-T scanners	Axial and coronal FS-T2WI; Axial chemical shift T1WI; DWI with b = 800; Axial or Coronal FS-dynamic contrast enhanced T1WI (corticomedullary, late nephrographic, and excretory phases)
Ibrahim2023 [27]	MRI	Diverse imaging protocols in multiple institutions	Diverse 1.5-T and 3.0-T scanners	Axial and/or coronal T2WI; Axial chemical shift T1WI; Axial or Coronal FS-dynamic contrast enhanced T1WI (corticomedullary, and delay phases)
AlNasibi2022 [28]	CT	Institutional standard clinical imaging protocol	Diverse multiple detector CT scanners with 64-320 channel	An unenhanced acquisition of the kidneys; a corticomedullary phase acquisition of the kidneys; a nephrographic phase acquisition of the entire abdomen
Eldihimi2023 [29]	CT	Institutional standard clinical imaging protocol	Diverse multiple detector CT scanners with 64-320 channel	An unenhanced acquisition of the kidneys; a corticomedullary phase acquisition of the kidneys; a nephrographic phase acquisition of the entire abdomen
Lemieux2023 [30]	CT	Whether or not scans were performed using a renal mass protocol	Whether scans were performed at the study institution or at an outside institution	A corticomedullary phase, defined as the phase in which the renal cortex was the most avidly enhancing portion of the kidney

Note: ADC = apparent diffusion coefficient, ccLS = clear cell likelihood score, ccRCC = clear cell renal cell carcinoma, NR = not reported, T1WI = T1 weighted images, T1WI + C= T1 weighted images with contrast enhancement, FS-T2WI = fat-saturated T2 weighted images, T2WI = T2 weighted images, DWI = diffusion weighted images.

Supplementary Table S8 Rating process of included studies

Study [Reference]	No. of rater	Experience of rater	Training session	Blindness	Consensus method
Canvasser2017 [19]	1 of 7	Radiologists with fellowship training in body MRI from a single institution with a busy MRI service (approximately 7,000 abdominopelvic MRIs per year), with experience of 1 to 15 years	A refresher training session with a slide presentation, including examples of the main imaging features associated with ccRCC histology and other common histological diagnoses of renal masses	Blinded to final pathology results	Independent review
Johnson2019 [20]	1 of 14	Experienced academic radiologists covering the clinical service	NR	Prospectively assigned ccLS	Prospectively assigned ccLS
Steinberg2021 [22]	1 of 16	Fellowship-trained abdominal radiologists covering the clinical service	Had previously been trained in assigning ccLS	Prospectively assigned ccLS	Prospectively assigned ccLS
Dunn2022 [23]	3	Abdominal radiologists with experience of 7 to 12 years	provided with original research articles and review articles describing the application of the ccLS algorithm	Blinded to final pathology results	Independent review
Schieda2022 [24]	2 of 10	Radiologists with 5–30 year of post-fellowship experience	A 1.5-hour virtual teaching session where the senior author reviewed the use of the ccLS version 2.0, algorithm and presented several example cases	Blinded to clinical information including the histopathological diagnosis	Independent review
Hao2023A [25]	2	Radiologists with 3–15 year of post-fellowship experience	A training session of ccLS version 2.0	Blinded to clinical information including the histopathological diagnosis	Independent review
Hao2023B [26]	2 of 6	Radiologists with 1–15 year of post-fellowship experience	A training session of ccLS version 1.0 and 2.0	Blinded to clinical information including the histopathological diagnosis	Independent review
Ibrahim2023 [27]	2	Fellowship-trained abdominal radiologists with 9-20 year of post-fellowship experience	A 1-h session using the recently published step-by-step ccLS scoring guide and several example cases; provided with a summary diagram of the ccLS grading system in written and graphic format	Blinded to clinical information including the histopathological diagnosis	Independent review
AlNasibi2022 [28]	2	Fellowship-trained radiologists, both with 1 year of post-fellowship experience	Reviewed CT ccLS before evaluating the masses	Blinded to pathology results	Independent review
Eldihimi2023 [29]	3	Two fellowship-trained radiologists, with 12 and 17 years of post-fellowship experience, and an abdominal radiology fellow	Both radiologists were provided with a short presentation depicting the imaging features and how to apply them and the diagnostic algorithm	Blinded to pathology results	Independent review
Lemieux2023 [30]	5	Fellowship-trained abdominal radiologists with 2-7 years of post-fellowship experience,	Reviewed the initial study describing the proposed CT score as well as additional	NR	Independent review

		Abdominal imaging fellows, both of whom were in the first half of fellowship training	relevant literature describing other heterogeneity scores for renal mass evaluation		

Note: ccLS = clear cell likelihood score, ccRCC = clear cell renal cell carcinoma, NR = not reported.

Supplementary Table S9 QUADAS-2 assessment by two reviewers and consensus results

Study [Reference]	Risk of bias				Applicability concern		
	Patient selection	Index test	Reference standard	Flow and timing	Patient selection	Index test	Reference standard
Canvasser2017 [19]	L/L/L	L/L/L	L/L/L	U/U/U	L/L/L	L/L/L	L/L/L
Johnson2019 [20]	L/L/L	H/U/H	U/L/L	U/U/U	L/L/L	L/L/L	L/L/L
Steinberg2021 [22]	L/L/L	L/L/L	U/L/L	U/U/U	L/L/L	L/L/L	L/L/L
Dunn2022 [23]	L/L/L	U/H/U	U/L/L	U/U/U	L/L/L	L/L/L	L/L/L
Schieda2022 [24]	L/L/L	L/L/L	U/L/L	U/H/H	L/L/L	L/L/L	L/L/L
Hao2023A [25]	L/L/L	L/L/L	U/L/L	L/L/L	L/L/L	L/L/L	L/L/L
Hao2023B [26]	L/L/L	L/L/L	U/L/L	L/L/L	L/L/L	L/L/L	L/L/L
Ibrahim2023 [27]	L/L/L	L/L/L	U/L/L	U/U/U	L/L/L	L/L/L	L/L/L
AlNasibi2022 [28]	L/L/L	L/L/L	L/L/L	U/H/H	L/L/L	L/L/L	L/L/L
Eldihimi2023 [29]	L/L/L	L/L/L	U/L/U	U/H/H	L/L/L	L/L/L	L/L/L
Lemieux2023 [30]	L/L/L	H/H/H	L/L/L	U/H/H	L/L/L	H/H/H	L/L/L

The data were presented as First reviewer/ Second reviewer/ Consensus result. L = Low risk, U = Unclear, H = high risk.

Supplementary Table S10 Two-by-two data for meta-analysis

Study [Reference]	No. of mass	No. of ccRCC	No. of non-ccRCC	TP	FP	FN	TN	SEN	SPE	PPV	NPV	ACC
MRI ccLS												
Canvasser2017 [19]	121	61	60	*48*	*12*	*13*	*48*	78	80	NR	NR	79
Steinberg2021 [22]	213	120	93	103	39	17	54	86	58	84	96	74
Dunn2022 [23]	83	34	49	*29*	*9*	*5*	*40*	85	82	96	91	92
Schieda2022 [24]	250	119	131	*89*	*29*	*30*	*102*	75	78	76	77	NR
Hao2023B [26]	700	509	191	*404*	*28*	*105*	*163*	79.3	85.1	93.4	60.6	80.9
Ibrahim2023 [27]	61	25	36	*24*	*13*	*2*	*23*	94	63	65	94	NR
CT ccLS												
AlNasibi2022 [28]	148	78	70	*66*	*33*	*12*	*37*	85	53	NR	NR	70
Eldihimi2023 [29]	51	26	25	*20*	*10*	*6*	*15*	78	59	67	72	NR
Lemieux2023 [30]	97	43	54	*32*	*19*	*11*	*35*	74	65	59	90	NR

Note: ccLS = clear cell likelihood score, ccRCC = clear cell renal cell carcinoma. TP = histologically diagnosed clear cell renal carcinoma rated with a ccLS rating ≥ 4, FP = histologically diagnosed non-clear cell renal carcinoma rated with a ccLS rating ≥ 4, FN = histologically diagnosed clear cell renal carcinoma rated with a ccLS rating ≤ 3, TN = histologically diagnosed non-clear cell renal carcinoma rated with a ccLS rating ≤ 3. ccLS = clear cell likelihood score. SEN = sensitivity, SPE = specificity, PPV = positive likelihood value, NPV = negative likelihood value, ACC = accuracy. The reconstructed data were marked in *italic*.

Supplementary Table S11 Leave-one-out sensitivity analysis

Excluded study	Sensitivity	Specificity	Positive likelihood ratio	Negative likelihood ratio	Diagnostic odds ratio
MRI ccLS					
None (n = 6)	0.816 (0.776-0.850)	0.757 (0.668-0.828)	3.357 (2.463-4.575)	0.243 (0.204-0.290)	13.800 (9.458-20.135)
Canvasser2017 (n = 5)	0.820 (0.775-0.858)	0.747 (0.642-0.830)	3.244 (2.287-4.602)	0.241 (0.198-0.292)	13.484 (8.852-20.539)
Steinberg2021 (n = 5)	0.798 (0.770-0.824)	0.776 (0.685-0.846)	3.558 (2.477-5.110)	0.260 (0.219-0.308)	13.685 (8.349-22.432)
Dunn2022 (n = 5)	0.793 (0.762-0.821)	0.797 (0.738-0.845)	3.902 (2.992-5.089)	0.260 (0.222-0.304)	15.024 (10.327-21.858)
Schieda2022 (n = 5)	0.816 (0.755-0.865)	0.724 (0.632-0.800)	2.956 (2.248-3.887)	0.254 (0.197-0.327)	11.652 (8.028-16.911)
Hao2023B (n = 5)	0.800 (0.771-0.826)	0.748 (0.646-0.828)	3.169 (2.201-4.553)	0.268 (0.223-0.321)	11.845 (7.082-19.813)
Ibrahim2023 (n = 5)	0.829 (0.782-0.868)	0.752 (0.641-0.837)	3.343 (2.307-4.844)	0.227 (0.184-0.280)	14.716 (9.466-22.878)
CT ccLS					
None (n = 3)	0.799 (0.715-0.863)	0.587 (0.497-0.672)	1.937 (1.561-2.405)	0.342 (0.237-0.494)	5.666 (3.345-9.597)
AlNasibi2022 (n = 2)	0.827 (0.742-0.888)	0.547 (0.447-0.644)	1.827 (1.440-2.318)	0.316 (0.200-0.500)	5.778 (3.019-11.057)
Eldihimi2023 (n = 2)	0.754 (0.639-0.841)	0.633 (0.522-0.731)	2.053 (1.492-2.826)	0.389 (0.249-0.608)	5.274 (2.548-10.765)
Lemieux2023 (n = 2)	0.806 (0.706-0.877)	0.585 (0.478-0.684)	1.940 (1.524-2.469)	0.333 (0.218-0.508)	5.833 (3.247-10.480)

Note: ccLS = clear cell likelihood score.

Supplementary Figure S1 MRI clear cell likelihood algorithm version 2.0

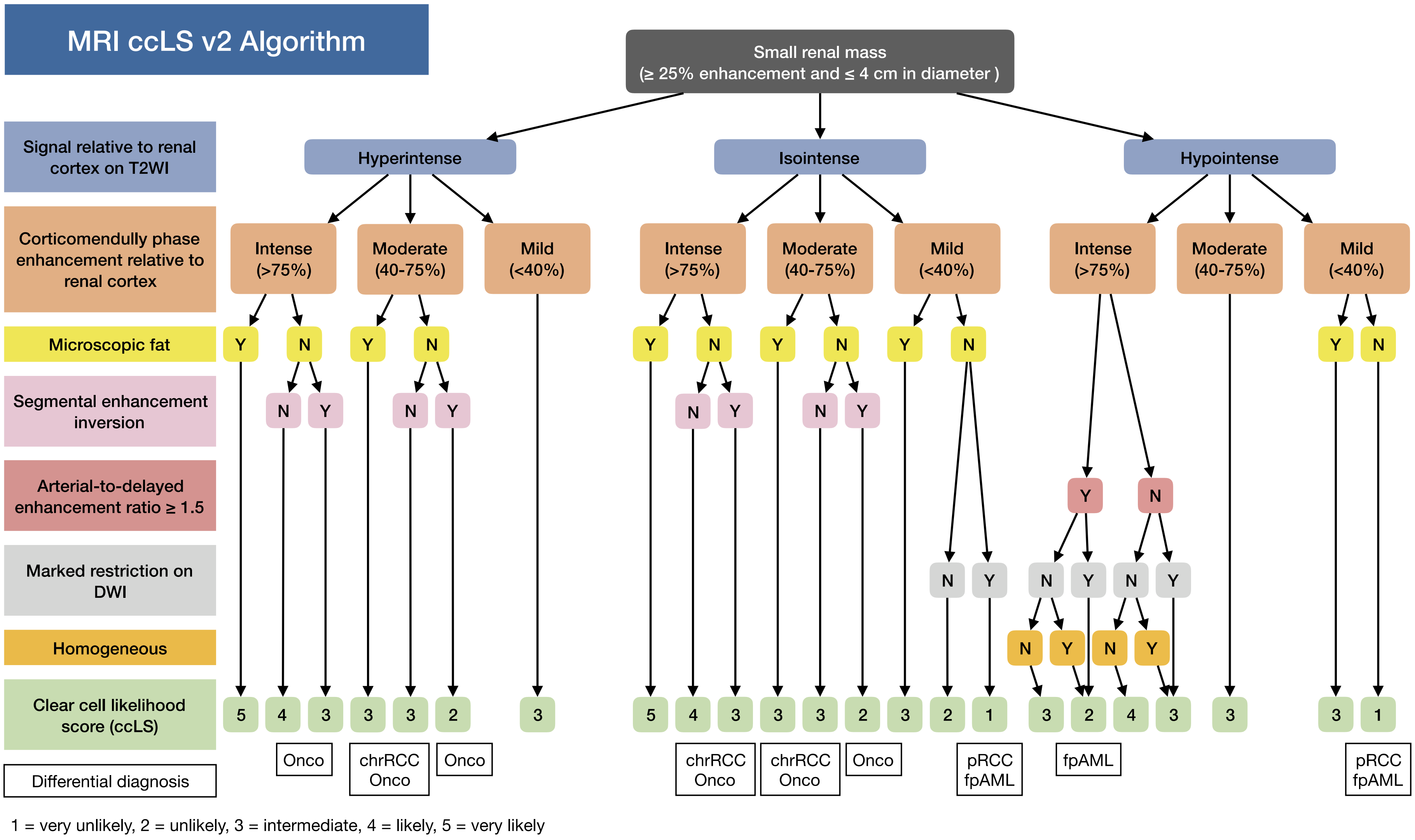

Note: ccLS = clear cell likelihood score, ccRCC = clear cell renal cell carcinoma, chrRCC = chromophobe renal cell carcinoma, pRCC = papillary renal cell carcinoma, fpAML = fat-poor angiomyolipoma, Onco = oncocytoma.

Insights Imaging (2024) Zhong JY, Hu YF, Xing Y, et al.

Supplementary Figure S2 CT clear cell likelihood algorithm

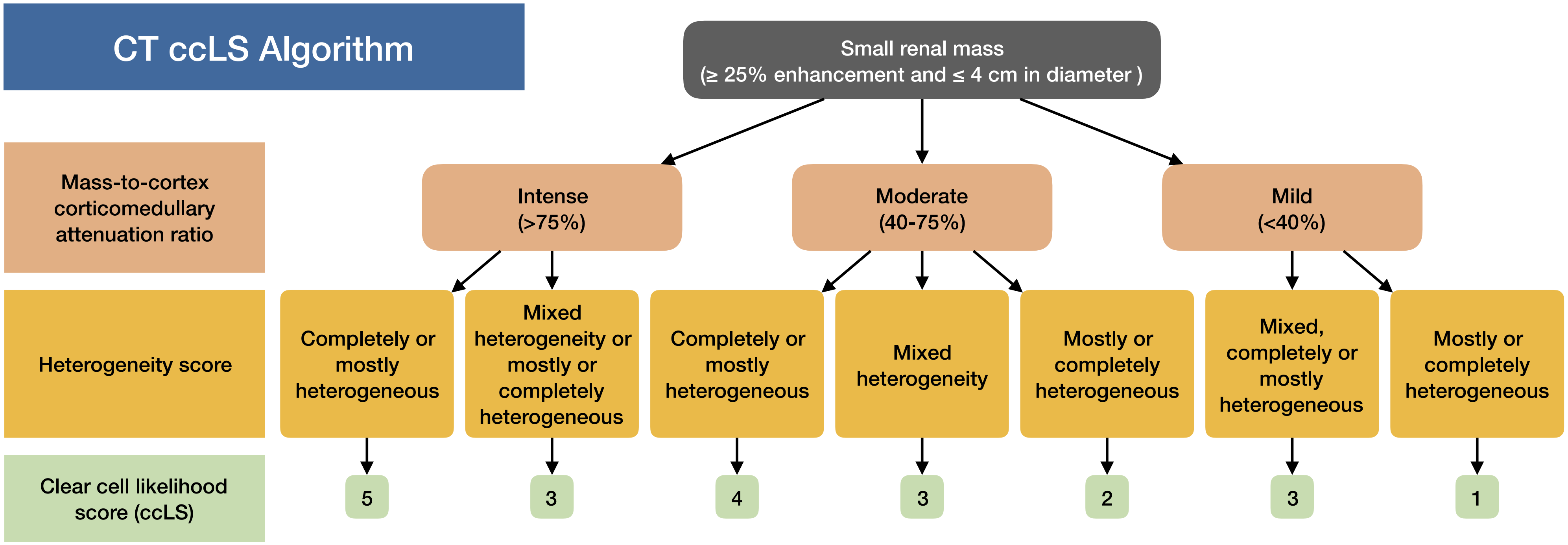

1 = very unlikely, 2 = unlikely, 3 = intermediate, 4 = likely, 5 = very likely
